# Altered brain structural covariance networks of the thalamic subfields in right chronic capsular stroke

**DOI:** 10.3389/fnins.2025.1650937

**Published:** 2025-09-26

**Authors:** Jun Guo, Hongchuan Zhang, Jingchun Liu, Caihong Wang, Chen Cao, Jingliang Cheng, Chunshui Yu, Wen Qin

**Affiliations:** ^1^Department of Radiology, Tianjin Huanhu Hospital, Huanhu Hospital Affiliated to Tianjin Medical University and Tianjin University HuanHu Hospital, Tianjin, China; ^2^Department of Radiology, Yijishan Hospital of Wannan Medical College, Wuhu, China; ^3^Department of Radiology, Tianjin Key Lab of Functional Imaging, Tianjin Institute of Radiology and State Key Laboratory of Experimental Hematology, Tianjin Medical University General Hospital, Tianjin, China; ^4^Department of Magnetic Resonance Imaging, The Fifth Affiliated Hospital of Zhengzhou University, Zhengzhou, China; ^5^Department of Magnetic Resonance Imaging, The First Affiliated Hospital of Zhengzhou University, Zhengzhou, China

**Keywords:** capsular stroke, gray matter volume, network Remoding, structural covariance network, thalamus subfield

## Abstract

**Background:**

The thalamus, along with its component nuclei, possesses extensive connections with various brain regions and is engaged in diverse functions. However, it is unknown whether the gray matter volume (GMV) covariance networks of thalamic subfields are selectively affected in chronic capsular stroke.

**Methods:**

We recruited 45 patients with chronic right capsular strokes (CS) and 93 normal controls (NC) from three centers. The thalamus was segmented into 25 subfields using FreeSurfer (v7.1.1). A general linear model was applied to investigate intergroup differences in the GMV covariance network of each thalamic subfield with each voxel of the entire brain between CS and NC, correcting for confounders such as age, gender, total intracranial volume (TIV), and scanners (voxel-wise *p* < 0.001, cluster-wise FWE corrected *p* < 0.05).

**Results:**

Our findings revealed that all 25 ipsilesional thalamic subfields in CS were atrophied (*p* < 0.05, FDR correction). Among these, 16 ipsilesional thalamic subfields (including AV, LD, LP, VLa, VLp, VPL, VM, CeM, CL, MDm, LGN, PuM, PuI, CM, Pf, and Pt) exhibited significantly subfield-specific increased GMV covariance connectivity with the anterior orbital gyrus, superior occipital gyrus, calcarine, anterior cingulate cortex, precentral gyrus, and other regions. Additionally, although none of the contralesional thalamic subfields demonstrated regional GMV changes, 3/25 showed subfield-specific increased GMV covariance connectivity with the ipsilesional anterior orbital gyrus and subcortex.

**Conclusion:**

The GMV covariance networks of thalamic subfields are selectively involved in patients with chronic capsular stroke, which affect not only the ipsilesional thalamic subfields but also the contralesional ones.

## Introduction

The human thalamus is a brain structure composed of numerous highly specific nuclear groups. Different thalamic nuclei have diverse functions (such as processing speed, attention, and executive functions) and are connected to different regions of the cerebral cortex, there is great interest in the neuroimaging community to study their volume, shape, and connectivity (Corona et al., 2020; Iglesias et al., 2018). The thalamus and basal ganglia collectively constitute a vital component of the cortico-striato-thalamo-cortical (CSTC) circuit. The anatomical vulnerability of the lenticulostriate arteries makes capsular infarction the predominant type of ischemic stroke, which usually involves the integrity of the CSTC circuit (Herrero et al., 2002; Yan et al., 2022). In such instances, the thalamus typically experiences secondary degenerative alterations, which not only compromise its structural integrity but also significantly modify its functional state, potentially resulting in motor and cognitive impairments (Ilves et al., 2022; Steiner et al., 2022). For example, some studies have shown that capsular stroke (CS) can lead to frontal dysfunction or memory impairment (Pantoni et al., 2001). The symptoms of frontal dysfunction are thought to be caused by disrupted connections between the thalamus and the frontal cortex (Biesbroek et al., 2017; Tatemichi et al., 1992; Zhang et al., 2021). This phenomenon may be ascribed to secondary degeneration of the thalamus, and this process is not confined solely to the ipsilesional thalamus, with the potential for involvement of the contralateral thalamus as well (Carey et al., 2011; Carmichael et al., 2004; Yassi et al., 2015).

While capsular strokes impact the adjacent thalamus, research often treats the thalamus as a single unit (Liu et al., 2019). However, the thalamus is highly heterogeneous, with distinct subfields differing in anatomical structure and function (Beloozerova and Marlinski, 2020; Lambert et al., 2017; Li et al., 2023). Each subfield has unique connections to the cortex, mediating specific functions. For example, the ventral lateral anterior (VLa) nucleus, part of the motor thalamus, connects with the substantia nigra and prefrontal cortex and functions as motor planning and learning (Kumar et al., 2014); The mediodorsal (MD) nucleus connects with the medial temporal and dorsolateral prefrontal cortex, and pallidum, and is involved in working memory and sensorimotor integration (Lambert et al., 2017). Critically, the integrity of the thalamocortical connection between the MD and dorsolateral prefrontal cortex is crucial for motor recovery post-stroke (Choi et al., 2020). Given this functional divisions of the thalamic subfields within the cortico-striato-thalamo-cortical (CSTC) circuit (Haber and McFarland, 2002; Mair et al., 2022), analyzing the entire thalamus’s volume obscures the specific cognitive and sensorimotor deficits caused by stroke.

Structural covariance network (SCN) is a valuable metric for investigating the brain’s topological organization, as it reflects the coordinated development and synchronous impact of interconnected regions (Liu et al., 2023; Zhang et al., 2025; Zielinski et al., 2010). This SCN is closely linked to cognitive abilities (Khundrakpam et al., 2017), genetic variant (Wen et al., 2023) and expression (Romero-Garcia et al., 2018) and neuropsychiatric disorders including stroke (Liu et al., 2023; Zhang et al., 2025). Therefore, examining both thalamic subfieldal structure and the structural covariance between thalamic subfields and remote cortical areas can improve our ability to identify the specific circuits disrupted by capsular stroke. This, in turn, may provide avenues for targeted rehabilitation.

In this context, we hypothesized that gray matter volume (GMV) covariance patterns in thalamic subfields would be selectively altered in patients with chronic capsular stroke. To test this hypothesis, we enrolled 45 patients with right internal capsule stroke and 93 healthy controls. We investigated whether the GMV covariance of thalamic subfields differed significantly between the two groups. Furthermore, we explored the potential associations between GMV covariance in these thalamic subfields and clinical behavior. This study aims to enhance our understanding of the GMV covariance alterations pattern of different thalamic subfields caused by capsular stroke and its subsequent functional consequences.

## Methods

### Subjects

The MRI data were acquired from three hospitals using three scanners. All enrolled patients had to meet the following criteria: firstly, it must be a first-time ischemic stroke and exhibited motor dysfuncion at stroke onset; secondly, the lesion involved the right internal capsule and was a single lesion; furthermore, the time interval between stroke onset and enrollment should exceed 6 months; In addition, right-handedness before the stroke. The exclusion criteria for subjects include: firstly, patients with a clinical history or confirmed recurrent stroke through imaging examination; **t**he second is the presence of other intracranial abnormalities, such as tumors, vascular malformations, etc.; thirdly, histories of other mental illnesses or drug dependence; Fourth, left-handedness before stroke. Finally, 45 patients with chronic right CS and 93 normal controls (NC) were enrolled for the research. The demographic data of participants are described in Table 1.

**Table 1:** Demographic and clinical information pertaining to patients with stroke and normal controls.

Variables	Capsular stroke	Normal controls	Statistics	*p*-value
Age (year)	55.51 ± 7.31	55.53 ± 7.43	*t =* 0.012	0.990
Gender (male/female)	27/18	50/43	*χ2* = 0.478	0.489
FMA total	100 (82.5, 100)	100 (100, 100)	Z = −7.097	<0.001
RAVLT-SR	42.778 ± 10.396	49.527 ± 8.305	*t* = −3.807	<0.001
RAVLT-LR	11 (9, 12)	12 (10, 14)	*Z =* −2.25	0.024
N_ACC	0.90 (0.87, 0.95)	0.93 (0.88, 0.95)	*Z* = −1.082	0.279
N_RT	805.15 (726.42, 1003.88)	758.16 (666.18, 914.14)	*Z* = 1.617	0.106
S_ACC	0.92 (0.85, 0.95)	0.92(0.89, 0.95)	*Z* = −1.164	0.245
S_RT	849.72 (738.52, 1030.49)	794.86 (690.30, 929.56)	*Z* = 1.580	0.114
Lesion volume at chronic stage (mL)	243.796 ± 50.248	/	*/*	/

Continuous variables are presented as mean ± SD for normally distributed data, or as median (interquartile range) for non-normally distributed data. FMA, Fugl-Meyer assessment; N_RT, reaction time of number 1-back test; N_ACC, accuracy of number 1-back test; S_RT, reaction time of spatial 1-back test; S_ACC, accuracy of Spatial 1-back test; SD, standard deviation.

The experimental protocol was approved by the ethics committee of Tianjin Medical University General Hospital (IRB2015-092-02), Tianjin Huanhu Hospital (JinHuan 2022-103), and the first affiliated hospital of zhengzhou university (2018-KY-03), and written informed consent was obtained from all participants prior to enrolment.

### Clinical behavioral assessment

All participants underwent behavioral assessments about motor and recognition functions. Fugl-Meyer assessment (FMA) was used to evaluate motor function of stroke patients. Rey Auditory Verbal Learning Test (RAVLT) used to assess verbal learning and memory, including short-term and long-term memory (Hochstenbach et al. 1998). The working memory (WM) test included number 1-back and spatial 1-back tasks. E-Prime software, developed by Psychology Software Tools in Pittsburgh, PA, USA, was utilized to measure the accuracy (ACC) and response time (RT) of the participants. The 1-back task is a continuous processing model that effectively evaluates WM function and is widely employed in neuroscience research. The detailed clinical data are described in Table 1.

### Structutral MR images acquisition

High-resolution structural MRI images were acquired using 3.0 T MR scanners at three hospitals, including two GE Healthcare Discovery MR750 scanners and one Siemens Magnetom TrioTim scanner. For MR750, a brain volume (BRAVO) sequence was applied with the following parameters: Repetition Time (TR)/Echo Time (TE)/Inversion Time (TI) = 8.14/3.17/450 ms, flip angle = 12°, field of view (FOV) = 256 mm × 256 mm, matrix = 256 × 256, thickness = 1 mm, slices = 188, voxel size = 1 mm × 1 mm × 1 mm; for TrioTim, a magnetization prepared rapid acquisition gradient echo (MPRAGE) sequence was used: TR/TE/TI = 2000/2.26/900 ms, flip angle = 9°, FOV = 256 mm × 232 mm, matrix = 256 × 232, thickness = 1 mm, and slices = 192, resulting in a 1 mm isotropic voxel.

### Segmentation of thalamic subfields

**Figure 1:**
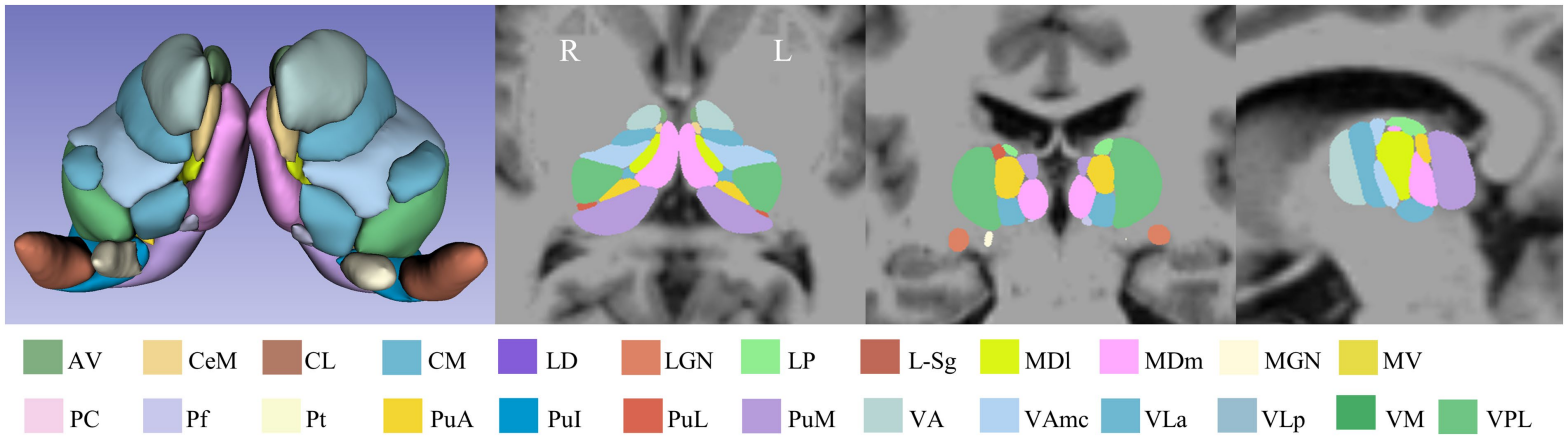
Schematic overview of the thalamic subfield segmentations for one NC subject. L: left, R: right.

Thalamic subfields were segmented using FreeSurfer software v7.1.1.^1^ The thalamic subfields module in FreeSurfer was used to perform automated segmentation of the thalamus, based on probabilistic atlases derived from histological data. This segmentation used each subject’s high-resolution T1 structural images and was processed with a Bayesian algorithm through the recon-all pipeline. This approach successfully segmented the thalamus into 25 distinct subfields per hemisphere (Iglesias et al., 2018; Manmatharayan et al., 2023). Each subfield was automatically labeled, and its volume was extracted. The total volume of the bilateral thalamus and the total intracranial volume were also derived. Figure 1 illustrates the thalamic subfield segmentations for a representative healthy control subject.

### Voxel-based morphometry of the whole brain

High-resolution structural MRI images were further preprocessed using a VBM pipeline with the CAT12.7 (r1742) toolbox^2^ integrated within SPM12^3^ in MATLAB 2016b to derive gray matter volume (GMV). Key steps included: (1) bias-field correction to ensure image singal uniformity; (2) tissue segmentation to distinguish between gray matter, white matter, and cerebrospinal fluid; (3) normalization to MNI space using the DARTEL algorithm for standardization across subjects; (4) resampling to a resolution of 1.5 × 1.5 × 1.5 mm; and (5) smoothing of gray matter maps using a 6 mm FWHM Gaussian kernel to enhance the signal-to-noise ratio and minimize local variations.

### The stroke lesion probability map calculation

**Figure 2:**
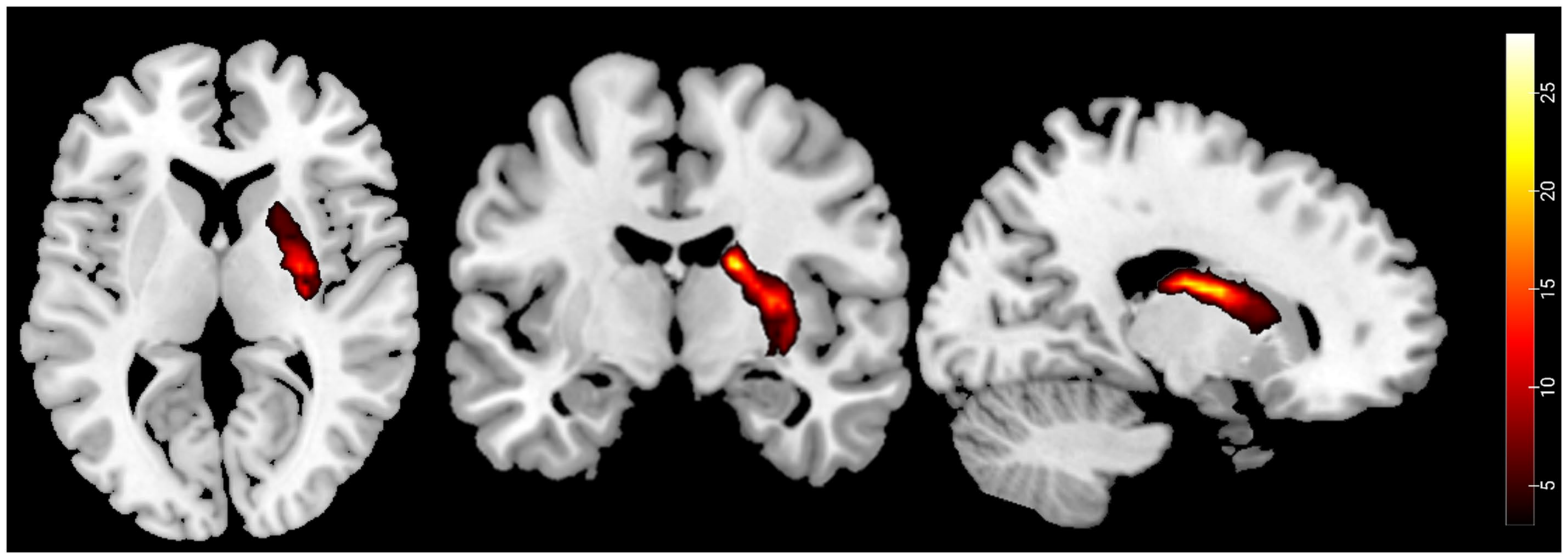
Probability maps of capsular stroke lesions. Color bar represents the lesion probability across patients.

First, an experienced neuroradiologist used MRIcron software to precisely identify and manually delineate each stroke patient’s lesion on the original T1-weighted images, creating a customized lesion mask for each patient. Second, these T1-weighted images were spatially normalized to MNI space. The lesion masks were then co-registered to MNI space using the normalization parameters. Finally, a group-level lesion probability map was generated by combining all lesion masks in MNI space and dividing by the number of patients. The stroke lesion probability map is shown in Figure 2.

### Statistical analyses

#### Demography and clinical statistics

One-sample Kolmogorov Smirnov test was used to evaluate the distribution of continuous data. Two-sample *t*-test (normal distribution) or the Mann–Whitney U test (non-normal distribution) were used to compare inter-group differences for continuous variables. Chi-square test was used for categorical variables including gender (*p* < 0.05).

#### Inter-group differences in SCN between thalamic subfields and each brain voxel

To estimate and compare the GMV covariance connectivity (SCN) between CS and NC, a general linear model (GLM) implemented in SPM12 was used with the Equation 1:


(1) $$\begin{array}{l} \mathrm{GMV}=\beta_{1}*\text{Labe}l_{\mathrm{cs}}+\beta_{2}*\text{Labe}l_{\mathrm{nc}}+\beta_{3}*\text{SubfieldVolum}e_{\mathrm{cs}}\\ +\beta_{4}*\text{SubfieldVolum}e_{\mathrm{nc}}+\sum \beta_{x}*\text{Confounders}+\varepsilon \end{array}$$

Where Label represents the group identifier, such that $\beta_{1}$ and $\beta_{2}$ represents the mean GMV for CS and NC group, respectively. SubfieldVolume represents the *z*-scored volume of a thalamic subfield, and its coefficients ( $\beta_{3}$ and $\beta_{4}$ ) represent the covariance connectivity between the volume of this thalamic subfield and each voxel in the whole brain for the CS and NC groups, respectively. Age, gender, scanner type, and total intracranial volume (TIV) were included in the model as covariates. A *t*-test was used to compare differences in covariance connectivity (contrast: [ $\beta_{3}$ - $\beta_{4}$ ]) between the CS and NC groups, with a voxel-wise threshold of *p* < 0.001 and cluster-wise correction for multiple comparisons at *p* < 0.05 using the family-wise error (FWE) method. We also carried the same analysis on the whole thalamus to compare the sensitivity of the SCN changes detected by whole thalamus analysis and those by thalamic subfields ways. For all voxel-wise statistics, voxels falling within the stroke lesion of any patients were excluded.

#### ROI extraction and *post-hoc* statistics

To identify the specific patterns of covariance connectivity disruption within thalamic subfields in CS, we extracted the mean GMV values from cortical and subcortical regions showing altered covariance with these thalamic subfields. Key steps included: (1) generating a binary mask from the statistical map for each thalamic subfield, preserving voxels showing significant between-group differences in SCN; (2) creating a union mask by combining the binary masks of all thalamic subfields; (3) identifying overlapping voxels between the union mask and each region defined by the Automated Anatomical Labeling 3 (AAL3) atlas (Rolls et al., 2020), and calculating the overlapping ratio between the intersecting voxels and each AAL3 region; (4) identifying target regions of interest (ROIs) based on a criterion that the overlapping ratio exceeded 10%; and (5) for each subject, extracting the average GMV within each selected target ROI. Subsequently, a ROI-based general linear model (GLM) analysis was conducted the *post-hoc* analysis with the same model as Equation 1 (*p* < 0.05, Bonferroni correction).

#### Inter-group differences in regional GMV of thalamic subfields

Intergroup differences in regional GMV of thalamic subfields were also compared using GLM model, with group as the main effect, controlling for confouders such as age, gender, scanner type, and total intracranial volume (TIV) [*p* < 0.05, false discovery rate (FDR) correction].

#### Correlation between alterated GMV covariance connectivity and clinical metrics in CS patients

A Spearman partial correlation analysis was used to test the association between clinical features (RAVLT_SR, RAVLT_LR, N_ACC, N_RT, S_ACC, S_RT and FMA) and thalamic SCN, controlling for the effects of age, gender, and scanners and the clinical features (*p* < 0.05, FDR correction).

## Result

### Demographic and clinical information

The characteristics of the subjects were shown in Table 1. There were no significant differences in age (two-sample *t*-test, *t* = 0.012, *p* = 0.990) and gender (*χ*^2^ = 0.478, *p* = 0.489) between the CS and NC. A preponderance of stroke patients demonstrate a substantial recovery in their motor function [FMA quantile = (82.5, 100)]. In comparison to the NC group, the RAVLT-SR scores in the CS group exhibited a significant decrease (*t* = 3.807, *p* < 0.001). However, no inter-group differences were observed for the RAVLT-LR scores (*Z* = −2.25, *p* = 0.024), reaction time of number 1-back (*Z* = −1.617, *p* = 0.106), spatial 1-back task (*Z* = −1.580, *p* = 0.114), number 1-back (*Z* = −1.082, *p* = 0.279) and spatial 1-back (Z = −1.164, *p* = 0.245) tasks. There were significant differences in volume of TIV and WM between the CS and NC, and no significant difference in volume of GM and CSF between the two groups (Supplementary Figure 1).

### Inter-group SCN difference of thalamic subfields

**Figure 3:**
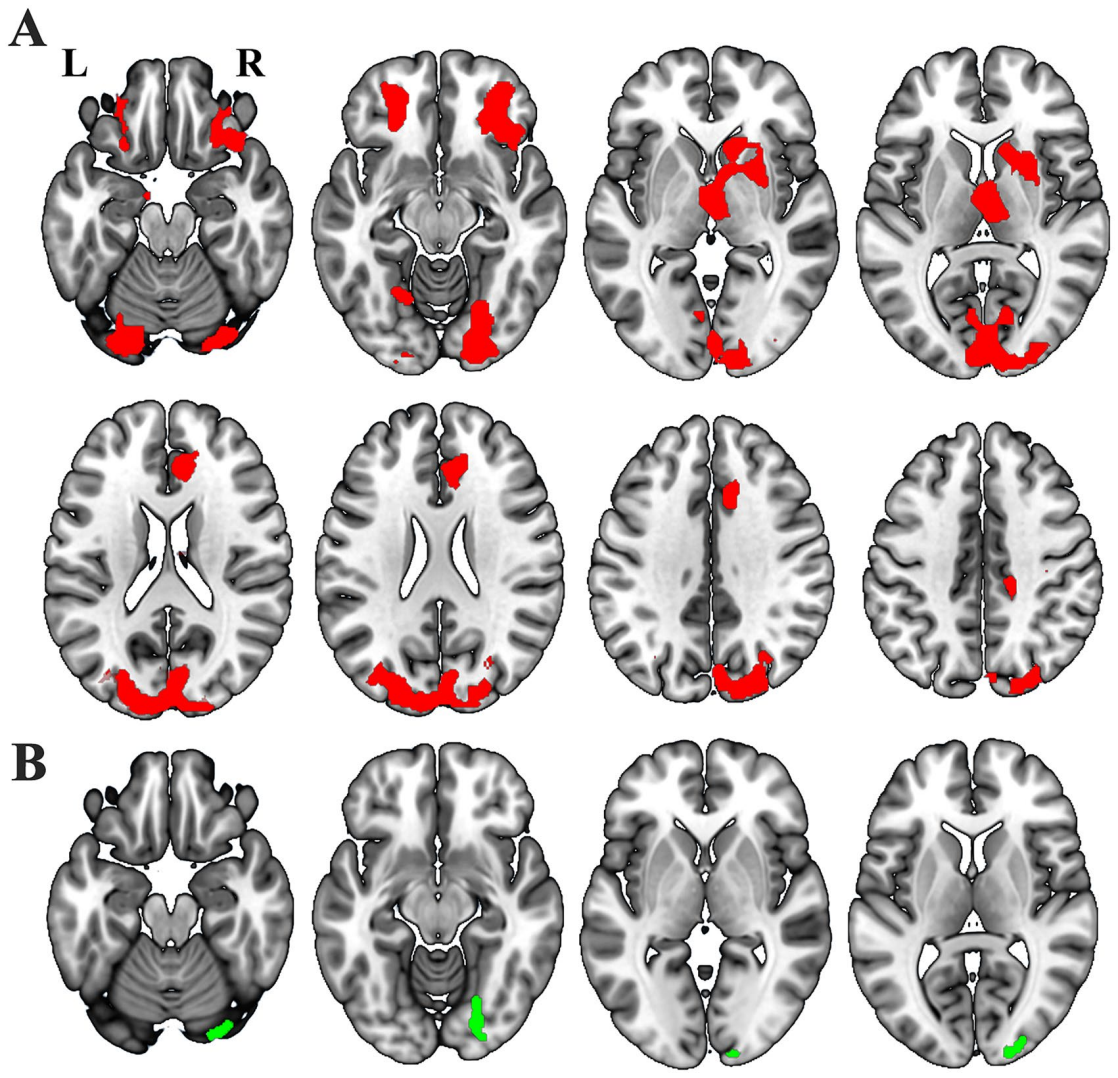
Different masks showthe notable GMV covariance results: thalamic subfields (**A**, red) and entire thalamus (**B**, green).

Voxel-wise GLM analysis revealed significant changed SCN between either one of the thalamic subfields and widespread of brain regions (Figure 3A), including the bilateral orbitofrontal cortex (OFC), bilateral dorsal and ventral visual cortex, ipsilesional thalamus, ipsilesional caudate, ipsilesional striatum, bilateral the anterior cingulate cortex (ACC), and so on. In contrast, increased SCN was only shown between the ipsilesional ventral visual cortex and the whole thalamus (Figure 3B) (*p* < 0.05, cluster-wise FWE correction).

**Figure 4:**
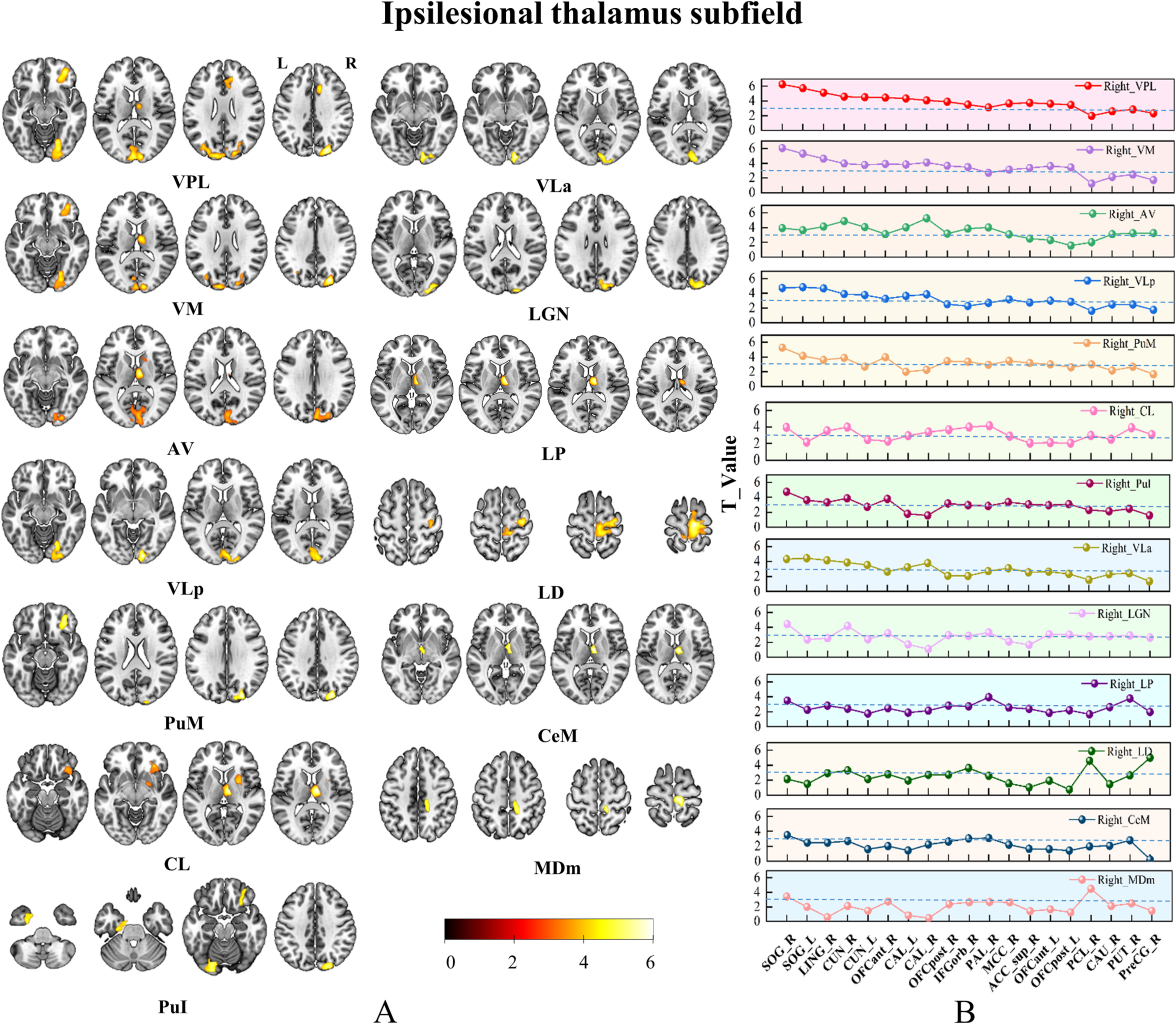
Brain regions with increased SCN in only ipsilesional thalamic subfields. Panel **(A)** Pshows the brain regions with changed SCN in each thalamic subfield. Color bar represents the T-values. **(B)** GMV covariance dot-line graph of the 13 thalamic subfields. The T-value of dot-line graph indicating inter-group differences in the covariance of GMV.

**Figure 5:**
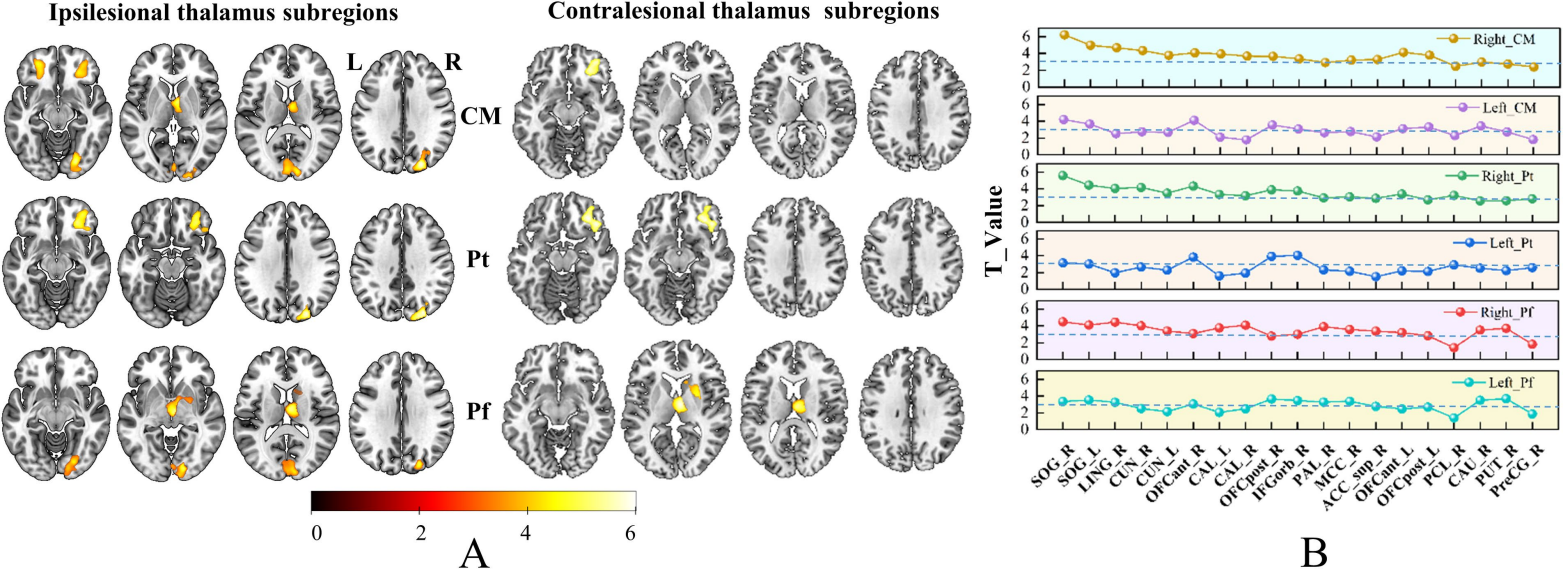
Brain regions with increased SCN in bilateral thalamic subfields. Panel **(A)** shows the brain regions with changed SCN in each thalamic subfield. Color bar represents the T-values. **(B)** GMV covariance dot-line graph of the six thalamic subfields. The T-value of dot-line graph indicating inter-group differences in the covariance of GMV.

We observed notably increased SCN for 16/25 ipsilesional thalamic subfields, including AV, LD, LP, VLa, VLp, VPL, VM, CeM, CL, MDm, LGN, PuM, PuI, CM, Pf and Pt (Figure 4A). Besides, we found 3/25 contralesional thalamic subfields showing increased SCN with the ipsilesional OFC, thalamus and striatum (Figure 5A).

### Subfield-specific SCN change patterns of CS patients

Based on the ROIs defined in the methods section, we selected 19 specific target regions. Dot-line graphs were created to illustrate the subfield-specific amplitudes of SCN changes (T-values) between each thalamic subregion and the 19 selected ROIs in CS patients (Figures 4B, 5B). Specifically, the ipsilesional SOG exhibited strengthened SCN with the ipsilesional VPL, VM, AV, VLa, LGN, and VLp, while the contralesional SOG showed strengthened SCN with the ipsilesional VPL and VM. The ipsilesional OFC demonstrated strengthened SCN with the ipsilesional VPL, VM, PuM, PuI, and CL. The ipsilesional ACC displayed strengthened SCN with the VPL. The ipsilesional medial thalamus showed strengthened SCN with the VPL, AV, LP, CL, and Cem, and the ipsilesional sensorimotor cortex (SMC) exhibited strengthened SCN with the ipsilesional LD and MDm.

### Subfields of thalamus GMV analysis

ROI-wise GLM analysis revealed a significant reduction in GMV across all 25 ipsilesional thalamic subfields in CS patients (Supplementary Figure 2) (*p* < 0.05, FDR correction). In contrast, no statistically significant GMV differences were observed in the contralesional thalamic subfields (Supplementary Figure 3) (*p* > 0.05, FDR correction).

### Association between the GMV covariance and clinical information

Spearman partial correlation analysis did not reveal any significant associations between thalamic SCN and clinical metrics after multiple comparison corrections (*p* > 0.05, FDR correction). However, using a looser threshold (*p* < 0.05, uncorrected), we found that spatial 1-back accuracy showed the highest number of weak associations with thalamic SCN in both ipsilesional (*N* = 19) and contralesional (*N* = 16) subfields in CS patients, followed by short-term memory performance on the RAVLT (*N* = 8 for ipsilesional, *N* = 16 for contralesional) (Supplementary Figure 4). On a subfield level, the ipsilesional L_Sg exhibited the highest number of weak associations (*N* = 6) with the clinical metrics, followed by the ipsilesional MGN (*N* = 5) (One L_Sg and one MGN were, respectively, associated with FMA). Additionally, the contralesional MGN, LD, and Pt also showed several weak associations (*N* = 4 for each subfield) with clinical metrics (Supplementary Figure 5 and Supplementary Tables 1–13). We also found that S_ACC showed the highest number of weak associations with thalamic SCN in right thalamic subfields (*N* = 15), in NC group, followed by N_ACC (*N* = 14). Additionally, some subfields of the left thalamus SCN also showed weak correlation with working memory (Supplementary Figure 6). On a subfield level, the right MGN exhibited the highest number of weak associations (*N* = 5) with the cognication metrics, followed by the right LGN, PuI, VLp (*N* = 4) (Supplementary Figure 7). The correlation between the right thalamic SCN and RAVLT_LR, N_ACC, N_RT, S_ACC, and S_RT also showed weak positive or negative associations between the CS and NC groups (Supplementary Figure 8). The correlation between the left thalamic SCN and RAVLT_LR also showed weak positive associations between the CS and NC groups (Supplementary Figure 9).

## Discussion

This study systematically investigates alterations in thalamic subfield structural covariance networks (SCNs) in patients with chronic right capsular stroke (CS), providing novel insights into thalamocortical reorganization mechanisms underlying post-stroke recovery. Utilizing high-resolution thalamic segmentation, we identified three key findings: (1) Subfield-level SCN mapping demonstrates superior sensitivity in detecting network-specific disruptions compared to conventional whole-thalamus approaches; (2) The GMV covariance networks of thalamic subfields are selectively involved in patients with chronic capsular stroke; and (3) contralesional thalamic subfields exhibit adaptive SCN remodeling despite preserved gray matter volume. These findings challenge the traditional whole-thalamus analytical framework and establish the critical role of subfield-specific network reorganization in stroke recovery, offering novel spatial-resolution perspectives for understanding neural plasticity, thereby enhancing our comprehension of the distinct functions played by thalamic subfields in the pathological course of CS.

The thalamus comprises functionally differentiated subfields, with distinct nuclei mediating specialized neural processes (Corona et al., 2020; Iglesias et al., 2018). The fundamental limitation of whole-thalamus analyses stems from the forced homogenization of inherently heterogeneous structures (Yuan et al., 2015), which obscures subfield-level functional antagonism or complementary patterns through artificial neutralization. While this simplification reduces analytical complexity, it critically compromises the understanding of fine-grained thalamic functional architecture and the pathophysiological mechanisms underlying thalamic-related disorder (Antonucci et al., 2021; Lin et al., 2018; McKenna et al., 2023; Yang et al., 2023). The high-resolution thalamic segmentation framework proposed by Iglesias et al. (2018) enables subfield-level structural covariance network (SCN) analysis, substantially enhancing our capacity to detect post-stroke thalamocortical network pathology. In our study, the whole-thalamus analysis identified only ipsilesional ventral visual cortex-thalamus SCN enhancement (Figure. 3B). In contrast, subfield-specific analysis revealed significant GMV covariance alterations in 16/25 ipsilesional and 3/25 contralesional thalamic subfields (Figures 4A, 5A), demonstrating the superior sensitivity of subfield-level approaches—consistent with prior studies showing that subfield decomposition uncovers otherwise hidden network changes (d'Ambrosio et al., 2017; Tung et al., 2022). Analogous methodology in schizophrenia research highlights the value of subfieldal decomposition—amygdala subfield analysis reveals more extensive GMV covariance abnormalities and distinct subfieldal engagement patterns, advancing understanding of limbic pathophysiology (Chang et al., 2024). These convergent findings underscore the critical advantage of subfield-level paradigms in elucidating network-level neuropathology.

While the thalamus does not receive direct vascular supply from the middle cerebral artery (MCA), accumulating evidence suggests that MCA infarction may induce secondary microstructural damage to the ipsilesional thalamus through remote degenerative mechanisms (Herve et al., 2005; Liu et al., 2019). Our findings extend these observations by demonstrating significant volumetric reduction of the ipsilesional thalamus in chronic capsular stroke patients, consistent with the thalamic atrophy patterns reported in prior studies (Liu et al., 2019; Wang et al., 2019). Through high-resolution thalamic parcellation into 25 distinct subfields, we further revealed that this volume loss occurred diffusely across all thalamic subfields compared to healthy controls. This whole thalamic degeneration pattern implies that secondary thalamic damage following capsular stroke may involve more extensive disconnection mechanisms than previously recognized (Herve et al., 2005; Liu et al., 2019; Wang et al., 2019), potentially disrupting both corticothalamic and thalamostriatal pathways (Haber and Calzavara, 2009; Lambert et al., 2017). The specific vulnerability of thalamic nuclei to retrograde degeneration might stem from their pivotal role as multimodal integration hubs within distributed cortical–subcortical networks (Hwang et al., 2017; Sherman, 2016; Tu et al., 2023).

Despite this widespread atrophy of thalamic subfields, SCN enhancements were selective: 16 ipsilesional subfields (e.g., VPL, VM, PuM) showed increased covariance with specific cortical regions, suggesting that structural covariance remodeling is not a random response but a targeted adaptation to preserve critical functions. We speculated that this may be related to the location of the lesions (Guo et al., 2019; Jiang et al., 2017; Wang et al., 2019). We observed that different thalamic subfields could demonstrate covariation with the same cerebral cortex.

For instance, ipsilesional thalamic subfields (VPL, VM, AV, VLa, LGN, and VLp) exhibited strengthened SCN with the superior occipital gyrus (SOG)—a region central to visual processing and spatial attention (Hageter et al., 2023; Reinhold et al., 2023). These subfields are key nodes in somatosensory, cognitive, visual, and motor pathways (Choi et al., 2022; Lambert et al., 2017), their enhanced covariance with the SOG likely reflects cross-modal compensation: as ascending sensory pathways are disrupted by capsular lesions (Herrero et al., 2002), residual connections between thalamic subfields and the SOG may be reinforced to maintain visual-somatosensory integration. Similarly, the ipsilesional OFCant also exhibited increased SCN with several thalamic subregions, such as VPL, VM, PuM, PuI, CM, and Pt. As research conducted by Bechara et al. (2000) has demonstrated, the OFC plays a vital role in decision-making and emotional regulation. This pattern suggests adaptive remodeling of thalamocortical loops supporting cognitive-emotional functions, consistent with prior findings that thalamo-cortical connectivity preserves affective stability post-stroke (Steiner et al., 2022). Furthermore, the strengthened SCN between the ipsilesional sensorimotor cortex (SMC) and the ipsilesional LD and MDm is also notable. According to previous studies, the MDm are associated with motor control and integration of sensory and motor information (Choi et al., 2020; Stockert et al., 2023).

In striking contrast to the ipsilesional thalamus, contralesional subfields showed no significant volume changes but exhibited enhanced SCN in 3/25 subfields—specifically with the ipsilesional OFCant, thalamus, and striatum. This dissociation between structural volume and covariance dynamics highlights a unique form of neural plasticity: the contralesional thalamus, though structurally intact, reorganizes its network connections to compensate for ipsilesional deficits. This pattern is best explained by the “diaschisis” effect—a phenomenon where focal brain lesions induce functional or structural changes in remote, interconnected regions (Carmichael et al., 2004; Seitz et al., 1999). Previous study (Yassi et al., 2015) demonstrated that contralesional thalamic microstructure is altered post-stroke, linked to functional disconnection of ipsilateral cortical regions; our findings extend this by showing that such changes manifest as enhanced structural covariance with the ipsilateral OFCant. Specifically, the contralesional thalamus may leverage interhemispheric projections (e.g., via the corpus callosum) to assume a “relay” role: as direct pathways from the ipsilesional thalamus to OFCant are damaged, contralesional subfields strengthen indirect connections to sustain critical cognitive-emotional functions. This is supported by Dermon and Barbas (1994) who identified robust contralateral thalamic projections to orbital cortices in non-human primates, providing an anatomical substrate for such cross-hemispheric compensation.

Previous animal studies have demonstrated that the majority of thalamic nuclei project into one or more well-defined cortical areas (Jones, 1990; Wise et al., 1996). The connection between the thalamus and the cortex is not a simple one-to-one relationship; rather, multiple cortical regions receive input from a single thalamic nucleus and transmit information back to different thalamic nuclei (Percheron et al., 1996). Hwang’s research also reveals significant functional connections between each nucleus and a minimum of three or more functional networks, and numerous cortical functional networks also demonstrate robust functional connections with multiple thalamic nuclei (Hwang et al., 2017). In our study, we found that some thalamic subfields exhibited increased GMV covariance with different cortical regions, which not only belonged to different functional networks, but also spanned to the contralesional hemisphere. For example, the strengthened SCN between the ipsilesional VPL and VM and bilateral SOG and ipsilesional OFCant. This may be the fact that thalamic nuclei play important roles in various cortical systems, and there seems to be overlapping patterns of connections among certain nuclei (Sherman and Usrey, 2024; Yuan et al., 2015). This overlap results in heterogeneity in the functional characteristics of individual nuclei, further compounding the complexity of thalamic nuclei functional representations (Sherman, 2007, 2016). Overall, these findings highlight the differential vulnerability and plasticity of thalamocortical circuits at the subfield level, providing valuable insights into the neural mechanisms underlying the observed structural changes.

In this study, after applying multiple comparison corrections, no significant correlation was identified between altered GMV covariance and clinical metrics in CS patients and the NC group—likely due to limited clinical variability: CS patients exhibited generally good functional recovery and mild symptoms, while NC group had no cognitive impairments, and this reduces the range of clinical measures needed to detect strong correlations. However, adopting a less stringent threshold (*p* < 0.05, uncorrected) revealed several intriguing yet weak associations. In CS patients, S_ACC showed the highest number of these weak associations with thalamic SCN: 19 in ipsilesional thalamic subfields and 16 in contralesional subfields, followed by RAVLT_SR with 8 associations in ipsilesional subfields and 16 in contralesional ones. This asymmetry reflects stroke-induced reliance on the ipsilesional thalamus for spatial working memory, while contralesional subfields contribute more to verbal memory. At the subfield level, the ipsilesional L_Sg was the top node (*N* = 6), followed by the ipsilesional MGN (*N* = 5), while the contralesional MGN, LD, and Pt each had 4 associations—indicating that CS patients rely disproportionately on ipsilesional subfields to sustain cognitive function, with contralesional subfields playing a secondary compensatory role. The NC group exhibited S_ACC had the weak associations with right thalamic SCN (*N* = 15), followed by N_ACC (*N* = 14), with left thalamic subfields also showing weak working memory links. Unlike CS group, NCs exhibited a right-weighted but non-lateralized pattern. Additional, the NC group also shared weak associations with CS patients for key metrics (e.g., right thalamic SCN with RAVLT_LR, working memory indices; left thalamic SCN with RAVLT_LR), indicating preserved fundamental thalamic-cognitive links. These cross-group findings collectively suggest that while no associations reached strict statistical significance, certain cognitive measures (especially spatial working memory) exhibit subtle relationships with specific thalamic subfields in both groups. The nuanced pattern of weak associations highlights the complexity of the relationship between thalamic subfields GMV covariance and behavior in CS patients, underscoring the need for more refined analytical approaches to fully elucidate these interactions. Future studies should explore potential confounding factors and consider longitudinal designs to better understand the temporal dynamics of these associations.

## Limitation

Several limitations of our study should be considered. First, our patient cohort exhibits high homogeneity, characterized by nearly complete motor recovery (median FMA = 100, interquartile range [82.5, 100]) and mild cognitive deficits. This is likely due to practical barriers in recruiting chronic stroke patients with moderate-to-severe deficits, who often cannot complete MRI scans or standardized assessments. The low variability in clinical measures weakened our ability to detect associations with SCN changes, as statistical power for correlation analyses depends on sufficient range in both variables. Furthermore, this sample bias limits generalizability: our findings may specifically reflect SCN remodeling in well-recovered chronic capsular stroke, rather than the broader stroke population (including those with persistent motor/cognitive impairments). Thus, our results should be interpreted with caution and validated in cohorts encompassing diverse recovery outcomes. Second, this is a cross-sectional study, which constraining its capacity to monitor the temporal variations in the covariance patterns of GMV across thalamic subfields. Longitudinal studies are crucial for observing the volume changes in thalamic subfields in stroke patients, as well as the progression of covariance between these subfields and cortical structures. Moreover, they are also essential for establishing clearer causal relationships between thalamic subfield structural covariance patterns and clinical outcomes. Additional validation in cohorts encompassing greater symptom severity heterogeneity is warranted to confirm these findings. Finally, our current research focuses on the structural covariance characteristics of thalamic subfields, but the study of the causal relationship with the structural covariance of the cerebral cortex is still shallow. In the future, we will deepen the research on the causal structural covariance of thalamic subfields to more accurately reveal the mechanism of its structural covariance with the cerebral cortex after stroke. This is crucial for understanding the interaction between them and the impact of stroke.

## Conclusion

In conclusion, our research investigated the SCN of thalamic subfields in patients with capsular stroke for the first time. The GMV covariance networks of thalamic subfields, which are selectively involved in patients with chronic capsular stroke, affect not only the ipsilesional thalamic subfields but also the contralesional ones. These subfield-specific SCN disruptions likely reflect neurodegenerative cascades and compensatory plasticity mechanisms post-stroke, with implications for both motor and cognitive functional reorganization. Future research should prioritize longitudinal designs to delineate dynamic GMV covariance patterns across thalamic subfields and validate their clinical translatability as biomarkers of post-stroke.

## Data Availability

The original contributions presented in the study are included in the article/Supplementary material, further inquiries can be directed to the corresponding authors.

## Footnotes

^1^ https://surfer.nmr.mgh.harvard.edu/

^2^ http://www.neuro.uni-jena.de/cat/

^3^ http://www.fil.ion.ucl.ac.uk/spm/software/spm12/

## Glossary

ACCsup: supra callosal part of Anterior Cingulate Cortex
AV: anteroventral
CAL: calcarine
CAU: caudate
CeM: central medical
CL: central lateral
CM: centromedian
FWE: family-wise error
IFGorb: IFG pars orbitalis
LD: laterodorsal
LGN: lateral geniculate
LING: lingual
LP: lateral posterior
L-Sg: limitans (suprageniculate)
MDl: mediodorsal lateral
MDm: mediodorsal media
MCC: cingulate_mid
MGN: medial geniculate
MVre: reuniens (medial ventral)
N_ACC: number 1-back accuracy rate
N_RT: number 1-back response time
OFCant: anterior orbital gyrus
OFCpost: posterior orbital gyrus
PAL: pallidum
PC: paracentral
PCL: paracentral_lobule
Pf: parafascicular
PreCG: precentral gyrus;
Pt: paratenial
PuA: pulvinar anterior
PuI: pulvinar inferior
PuL: Pulvinar lateral
PuM: pulvinar medial
PUT: putamen
S_ACC: spatial 1-back accuracy rate
S_RT: spatial 1-back response time
SOG: superior occipital gyrus
VA: ventral anterior
VAmc: ventral anterior magnocellular
VLa: ventral lateral anterior
VLp: ventral lateral posterior
VM: ventromedial
VPL: ventral posterolateral
L: left
R: right
