# Supplementary material for: Altered brain structural covariance networks of the thalamic subfields in right chronic capsular stroke
Source: Front Neurosci. 2025 Sep 26;19:1650937. doi: 10.3389/fnins.2025.1650937 (PMC12498287; doi:10.3389/fnins.2025.1650937)
Supplement: Supplementary file 1 [file Data_Sheet_1.docx]

Altered brain structural covariance networks of the thalamic subfields in right chronic capsular stroke

Supplementary Materials


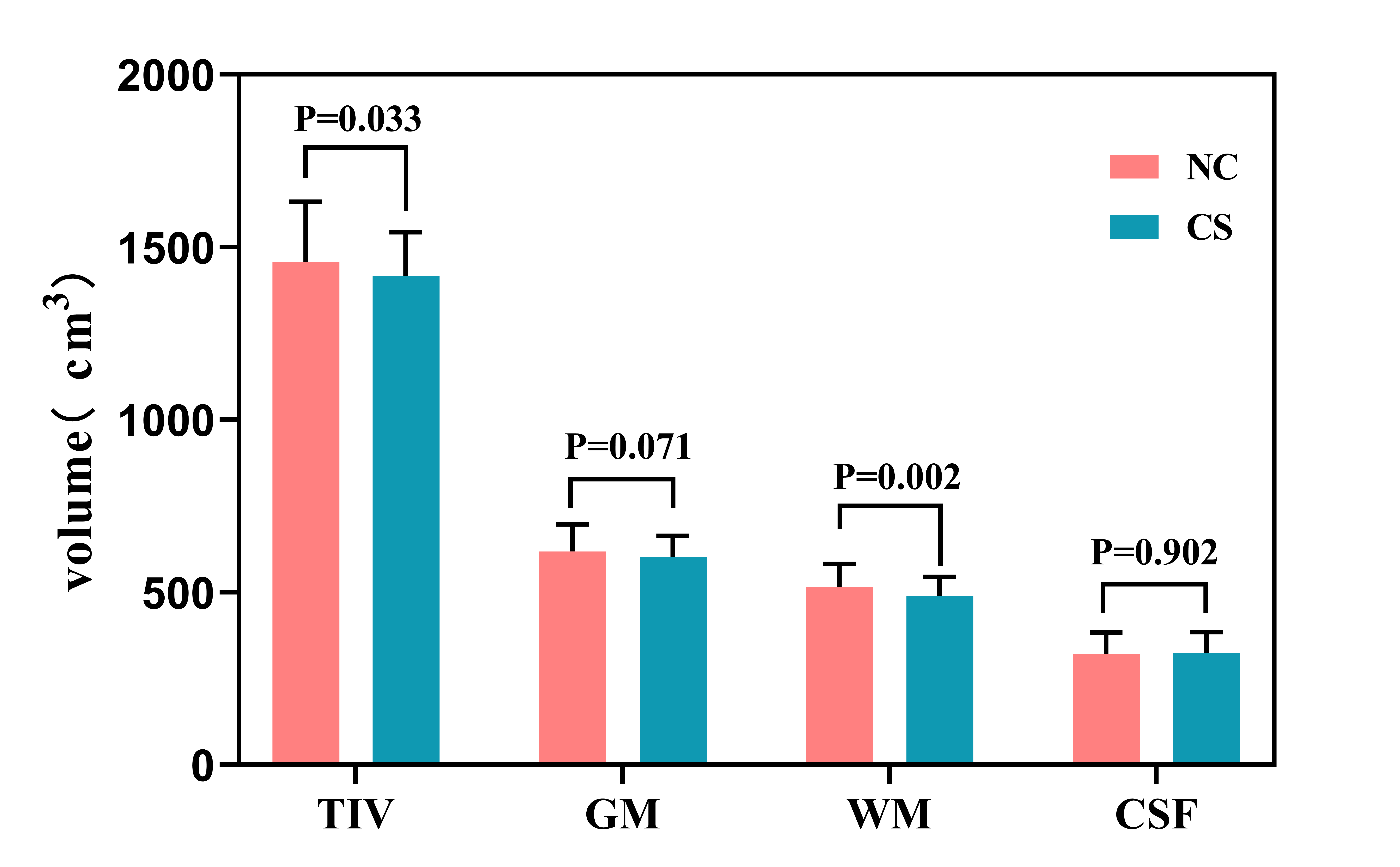


**Figure 1.** The volume of total intracranial (TIV), gray matter (GM), white matter (WM), and cerebrospinal fluid (CSF) in capsular stroke patients and normal controls groups(cm^3^)

**
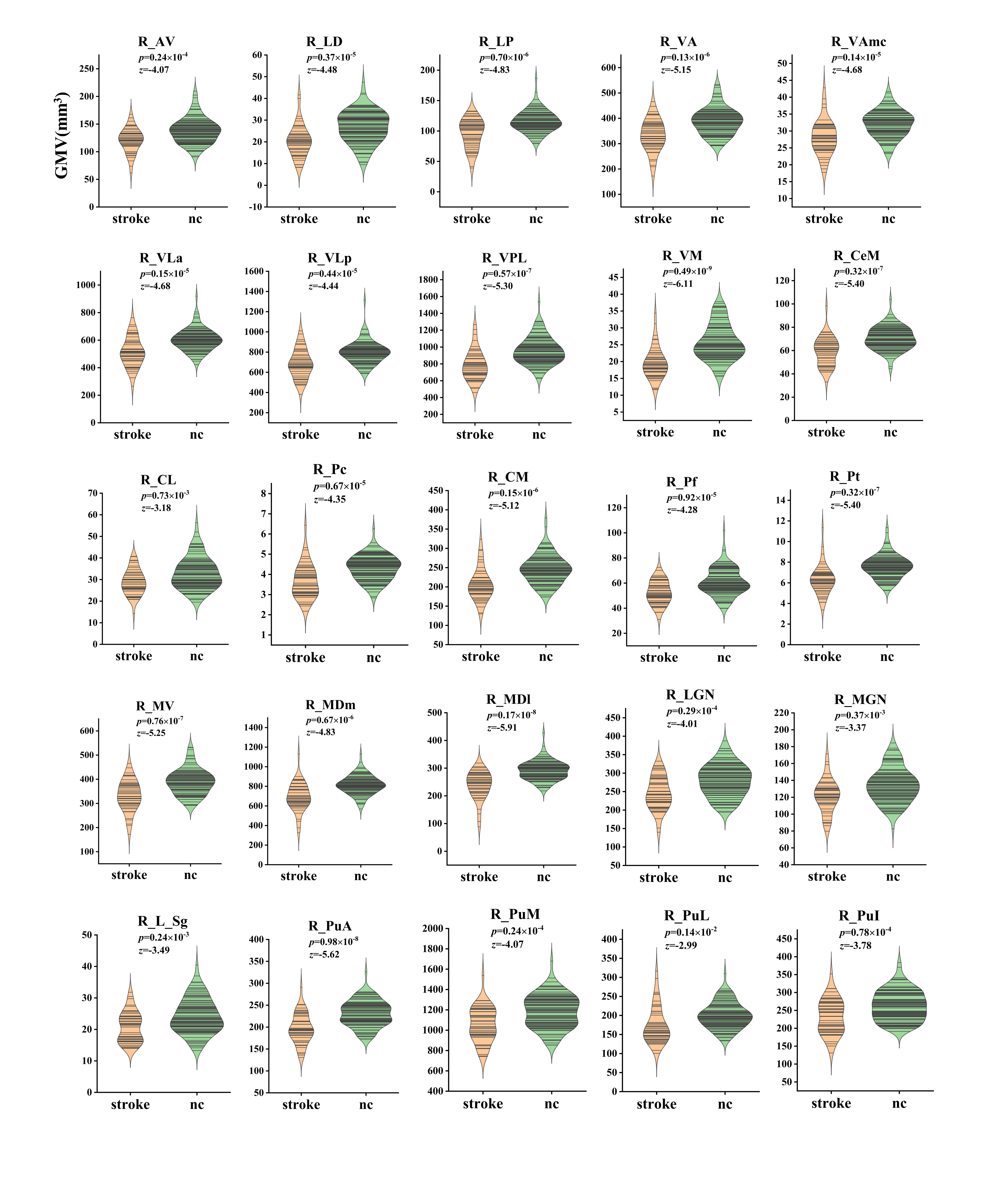
Figure 2.** The difference of volume of ipsilesional thalamic subfields between stroke patients and normal controls.

##### **
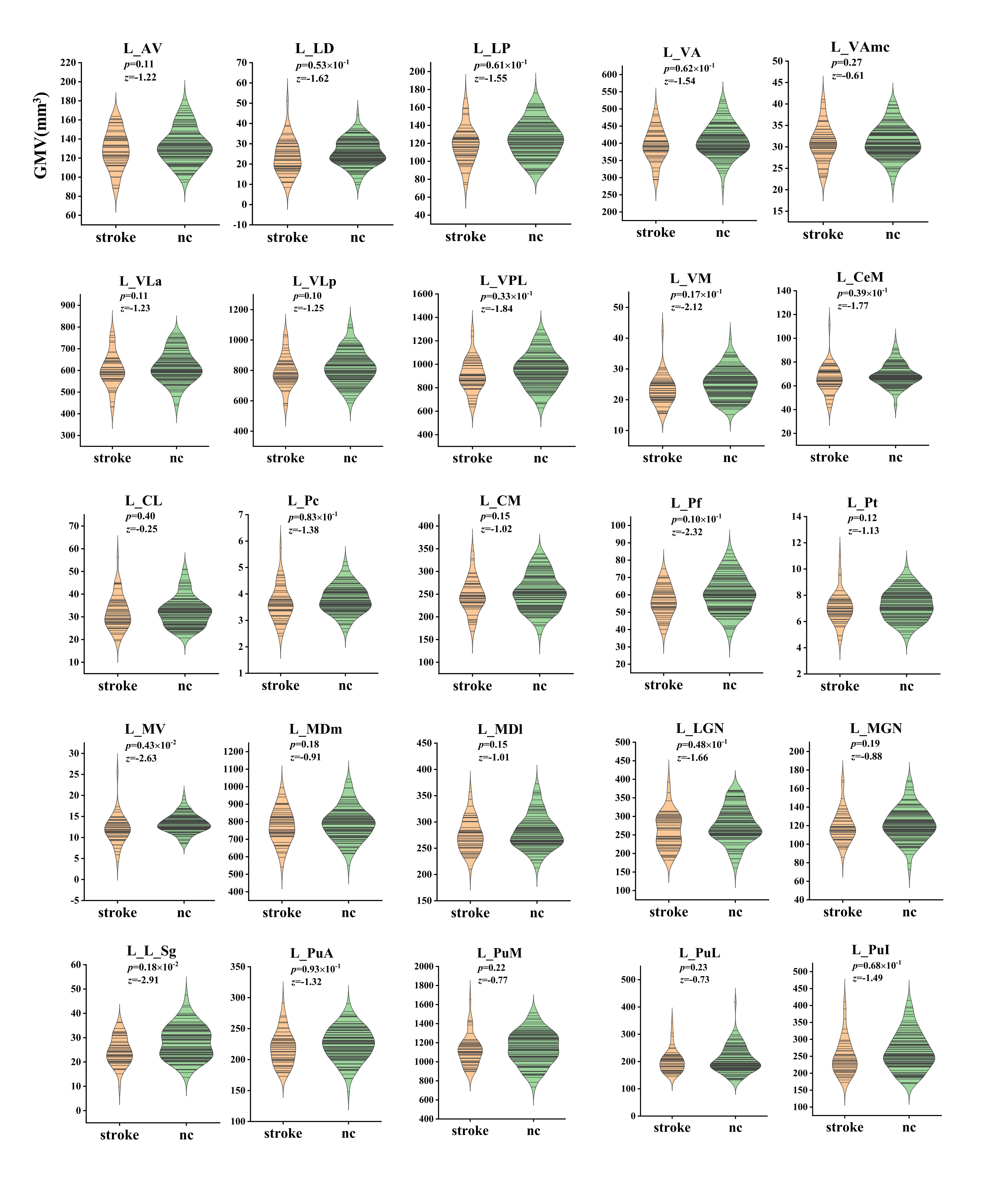
** Figure 3. The difference of volume of ipsilesional thalamic subfields between stroke patients and normal control.

## Table 1. Spearman correlation analysis of ipsilesional thalamic subfield covariance with RAVLT_SR.

| Targets | LGN | | MGN | | L_Sg | | MV | | CeM | | CL | | LP | | LD | |
| --- | --- | --- | --- | --- | --- | --- | --- | --- | --- | --- | --- | --- | --- | --- | --- | --- |
|  | r | p | r | p | r | p | r | p | r | p | r | p | r | p | r | p |
| IFGorb_R | 0.047 | 0.766 | -0.024 | 0.880 | -0.277 | 0.076 | 0.031 | 0.847 | 0.035 | 0.828 | 0.205 | 0.192 | 0.136 | 0.391 | 0.117 | 0.461 |
| OFCant_R | 0.137 | 0.387 | -0.021 | 0.897 | -0.030 | 0.849 | 0.311 | **0.045** | 0.345 | **0.025** | 0.369 | **0.016** | 0.425 | **0.005** | 0.344 | **0.026** |
| OFCpost_L | 0.386 | **0.012** | -0.076 | 0.633 | -0.388 | **0.011** | -0.150 | 0.344 | -0.097 | 0.543 | 0.209 | 0.184 | 0.173 | 0.275 | 0.215 | 0.172 |
| OFCpost_R | 0.093 | 0.558 | 0.036 | 0.820 | -0.188 | 0.232 | 0.051 | 0.747 | 0.083 | 0.601 | 0.176 | 0.266 | 0.136 | 0.391 | 0.088 | 0.579 |
| MCC_R | 0.025 | 0.877 | -0.160 | 0.310 | -0.049 | 0.756 | -0.057 | 0.722 | 0.004 | 0.980 | 0.229 | 0.144 | 0.204 | 0.194 | 0.295 | 0.058 |
| CAL_L | 0.183 | 0.246 | -0.187 | 0.237 | -0.216 | 0.170 | -0.121 | 0.446 | 0.001 | 0.995 | 0.220 | 0.162 | 0.134 | 0.396 | 0.213 | 0.175 |
| CAL_R | -0.040 | 0.803 | -0.145 | 0.358 | -0.050 | 0.754 | -0.193 | 0.222 | -0.184 | 0.244 | -0.059 | 0.709 | -0.089 | 0.575 | -0.033 | 0.835 |
| CUN_L | 0.085 | 0.594 | -0.023 | 0.887 | -0.120 | 0.449 | 0.000 | 0.999 | 0.048 | 0.763 | 0.187 | 0.236 | 0.110 | 0.489 | 0.245 | 0.117 |
| CUN_R | 0.061 | 0.701 | -0.123 | 0.439 | -0.074 | 0.644 | 0.020 | 0.898 | 0.018 | 0.911 | 0.152 | 0.336 | 0.084 | 0.597 | 0.240 | 0.125 |
| LING_R | 0.212 | 0.177 | 0.102 | 0.519 | 0.039 | 0.807 | 0.138 | 0.384 | 0.185 | 0.240 | 0.046 | 0.772 | 0.039 | 0.809 | 0.075 | 0.638 |
| SOG_L | 0.246 | 0.116 | 0.146 | 0.355 | 0.166 | 0.294 | 0.172 | 0.278 | 0.226 | 0.151 | 0.145 | 0.360 | 0.126 | 0.426 | 0.288 | 0.064 |
| SOG_R | 0.119 | 0.454 | -0.303 | 0.051 | -0.132 | 0.403 | -0.091 | 0.566 | -0.024 | 0.878 | 0.108 | 0.496 | 0.097 | 0.543 | 0.271 | 0.082 |
| PCL_R | 0.017 | 0.914 | -0.316 | **0.041** | -0.091 | 0.568 | 0.006 | 0.970 | 0.087 | 0.582 | 0.123 | 0.436 | 0.135 | 0.395 | 0.223 | 0.156 |
| CAU_R | 0.003 | 0.984 | 0.045 | 0.780 | -0.026 | 0.872 | 0.036 | 0.823 | 0.073 | 0.645 | 0.030 | 0.849 | 0.075 | 0.637 | -0.025 | 0.877 |
| PUT_R | -0.155 | 0.328 | -0.127 | 0.423 | -0.170 | 0.281 | -0.213 | 0.175 | -0.175 | 0.267 | -0.045 | 0.775 | -0.078 | 0.623 | -0.032 | 0.839 |
| PAL_R | -0.124 | 0.434 | -0.234 | 0.136 | -0.153 | 0.334 | -0.090 | 0.569 | -0.059 | 0.712 | 0.013 | 0.935 | -0.023 | 0.884 | 0.088 | 0.581 |
| ACC_sup_R | 0.059 | 0.712 | -0.145 | 0.359 | -0.033 | 0.837 | -0.031 | 0.845 | 0.019 | 0.906 | 0.203 | 0.198 | 0.209 | 0.184 | 0.097 | 0.543 |

Note: Black and bold represents P < 0.05, with underline represents P < 0.01.

Abbreviation: R=Right, L=Left, IFGorb= Frontal_Inf_Orb, ACC_sup= supra callosal part of Anterior Cingulate Cortex, CAL= Calcarine, CAU=Caudate, CUN=Cuneus, LING=Lingual, MCC=Cingulate_Mid, OFCant = Anterior orbital gyrus; OFCpost = Posterior orbital gyrus,PCL=Paracentral_Lobule, PUT= Putamen, PAL= Pallidum,PreCG=Precentral gyrus,SOG= Superior occipital gyrus

## Table 2. Spearman correlation analysis of ipsilesional thalamic subfield covariance with RAVLT_LR

| Targets | LGN | | PuI | | CM | | MV | | CeM | | PuL | | VLp | | LP | | LD | |
| --- | --- | --- | --- | --- | --- | --- | --- | --- | --- | --- | --- | --- | --- | --- | --- | --- | --- | --- |
|  | r | p | r | p | r | p | r | p | r | p | r | p | r | p | r | p | r | p |
| IFGorb_R | 0.168 | 0.289 | 0.202 | 0.199 | -0.020 | 0.899 | -0.051 | 0.748 | 0.004 | 0.982 | -0.245 | 0.118 | 0.035 | 0.827 | 0.099 | 0.531 | 0.145 | 0.359 |
| OFCant_R | 0.259 | 0.098 | 0.273 | 0.081 | 0.226 | 0.151 | 0.187 | 0.236 | 0.341 | **0.027** | -0.181 | 0.251 | 0.133 | 0.400 | 0.319 | **0.040** | 0.399 | **0.009** |
| OFCpost_L | 0.386 | **0.012** | 0.324 | **0.036** | 0.211 | 0.181 | 0.029 | 0.854 | 0.000 | 0.998 | -0.068 | 0.670 | 0.080 | 0.615 | 0.262 | 0.093 | 0.270 | 0.084 |
| OFCpost_R | 0.166 | 0.295 | 0.215 | 0.171 | -0.015 | 0.927 | 0.003 | 0.986 | 0.045 | 0.777 | -0.132 | 0.406 | 0.073 | 0.645 | 0.084 | 0.596 | 0.104 | 0.512 |
| MCC_R | 0.193 | 0.222 | 0.264 | 0.091 | 0.220 | 0.162 | 0.091 | 0.565 | 0.064 | 0.688 | -0.009 | 0.953 | 0.205 | 0.192 | 0.338 | **0.028** | 0.243 | 0.120 |
| CAL_L | 0.048 | 0.763 | 0.023 | 0.884 | 0.164 | 0.298 | 0.014 | 0.928 | 0.132 | 0.405 | -0.115 | 0.469 | 0.200 | 0.204 | 0.128 | 0.420 | 0.241 | 0.124 |
| CAL_R | -0.097 | 0.539 | -0.119 | 0.452 | 0.007 | 0.968 | -0.048 | 0.763 | -0.054 | 0.736 | -0.051 | 0.749 | 0.127 | 0.424 | -0.078 | 0.623 | 0.030 | 0.851 |
| CUN_L | -0.070 | 0.661 | -0.077 | 0.627 | 0.082 | 0.607 | 0.138 | 0.384 | 0.142 | 0.371 | -0.199 | 0.207 | 0.014 | 0.929 | 0.053 | 0.737 | 0.299 | 0.055 |
| CUN_R | 0.064 | 0.687 | 0.068 | 0.668 | 0.143 | 0.368 | 0.175 | 0.267 | 0.142 | 0.371 | -0.181 | 0.252 | 0.073 | 0.644 | 0.118 | 0.457 | 0.317 | **0.041** |
| LING_R | 0.197 | 0.212 | 0.136 | 0.390 | 0.098 | 0.537 | 0.136 | 0.391 | 0.093 | 0.558 | 0.104 | 0.514 | 0.144 | 0.365 | 0.070 | 0.660 | 0.065 | 0.681 |
| SOG_L | 0.185 | 0.241 | 0.000 | 0.999 | 0.357 | **0.020** | 0.311 | **0.045** | 0.362 | **0.019** | -0.029 | 0.855 | 0.195 | 0.217 | 0.103 | 0.517 | 0.328 | **0.034** |
| SOG_R | 0.163 | 0.303 | 0.029 | 0.853 | 0.371 | **0.016** | 0.069 | 0.665 | 0.172 | 0.278 | -0.038 | 0.812 | 0.335 | **0.030** | 0.215 | 0.171 | 0.274 | 0.079 |
| PCL_R | 0.177 | 0.263 | 0.035 | 0.828 | 0.160 | 0.311 | 0.043 | 0.787 | 0.194 | 0.219 | -0.335 | **0.030** | 0.106 | 0.506 | 0.174 | 0.270 | 0.156 | 0.324 |
| CAU_R | -0.054 | 0.737 | 0.065 | 0.682 | -0.058 | 0.715 | -0.014 | 0.932 | -0.049 | 0.759 | 0.018 | 0.912 | -0.077 | 0.627 | 0.108 | 0.494 | -0.141 | 0.373 |
| PUT_R | 0.046 | 0.772 | 0.166 | 0.294 | -0.092 | 0.560 | -0.169 | 0.284 | -0.209 | 0.185 | 0.026 | 0.868 | 0.027 | 0.865 | 0.141 | 0.373 | -0.093 | 0.558 |
| PAL_R | 0.048 | 0.763 | 0.113 | 0.477 | -0.006 | 0.970 | -0.045 | 0.776 | -0.095 | 0.549 | -0.081 | 0.612 | 0.017 | 0.916 | 0.154 | 0.331 | 0.027 | 0.866 |
| ACC_sup_R | 0.185 | 0.240 | 0.188 | 0.233 | 0.134 | 0.397 | -0.043 | 0.788 | -0.041 | 0.797 | 0.068 | 0.669 | 0.237 | 0.130 | 0.315 | **0.042** | 0.091 | 0.568 |

##### Note: Black and bold represents P < 0.05, with underline represents P < 0.01.

## Table 3(1). Spearman correlation analysis of ipsilesional thalamic subfield covariance with N_ACC

| Targets | L_Sg | | CM | | MDm | | Pf | | VAmc | | MDl | |
| --- | --- | --- | --- | --- | --- | --- | --- | --- | --- | --- | --- | --- |
|  | r | p | r | p | r | p | r | p | r | p | r | p |
| IFGorb_R | -0.145 | 0.360 | 0.003 | 0.984 | 0.019 | 0.906 | -0.074 | 0.643 | 0.057 | 0.722 | 0.080 | 0.616 |
| OFCant_R | 0.003 | 0.987 | 0.175 | 0.269 | 0.172 | 0.276 | 0.152 | 0.338 | 0.157 | 0.320 | 0.078 | 0.622 |
| OFCpost_L | -0.325 | **0.036** | 0.039 | 0.809 | -0.122 | 0.440 | -0.144 | 0.362 | -0.005 | 0.978 | 0.111 | 0.486 |
| OFCpost_R | -0.149 | 0.347 | -0.043 | 0.785 | 0.005 | 0.975 | 0.022 | 0.889 | -0.022 | 0.891 | 0.147 | 0.354 |
| MCC_R | -0.249 | 0.112 | -0.346 | **0.025** | -0.358 | **0.020** | -0.294 | 0.059 | -0.309 | **0.047** | -0.140 | 0.378 |
| CAL_L | -0.097 | 0.540 | -0.058 | 0.717 | -0.191 | 0.226 | 0.006 | 0.969 | -0.028 | 0.859 | -0.266 | 0.089 |
| CAL_R | -0.100 | 0.528 | -0.155 | 0.326 | -0.253 | 0.107 | -0.001 | 0.997 | -0.143 | 0.365 | -0.309 | **0.047** |
| CUN_L | -0.021 | 0.897 | 0.016 | 0.922 | -0.080 | 0.617 | 0.136 | 0.392 | 0.046 | 0.774 | -0.087 | 0.584 |
| CUN_R | 0.029 | 0.855 | -0.027 | 0.863 | -0.170 | 0.282 | 0.051 | 0.747 | -0.107 | 0.499 | -0.175 | 0.269 |
| LING_R | -0.043 | 0.786 | -0.104 | 0.514 | -0.296 | 0.057 | 0.030 | 0.852 | -0.300 | 0.054 | -0.192 | 0.224 |
| SOG_L | -0.032 | 0.843 | -0.001 | 0.996 | 0.051 | 0.746 | 0.202 | 0.200 | -0.050 | 0.754 | 0.056 | 0.725 |
| SOG_R | -0.121 | 0.444 | -0.163 | 0.301 | -0.129 | 0.417 | -0.146 | 0.355 | -0.188 | 0.232 | -0.190 | 0.228 |
| PCL_R | 0.066 | 0.679 | -0.134 | 0.398 | 0.129 | 0.415 | -0.244 | 0.120 | 0.064 | 0.689 | 0.140 | 0.378 |
| CAU_R | -0.079 | 0.618 | -0.143 | 0.366 | -0.169 | 0.286 | -0.194 | 0.218 | -0.213 | 0.176 | -0.036 | 0.819 |
| PUT_R | -0.126 | 0.426 | -0.276 | 0.077 | -0.255 | 0.103 | -0.396 | **0.009** | -0.321 | **0.038** | -0.146 | 0.356 |
| PAL_R | -0.165 | 0.296 | -0.244 | 0.119 | -0.249 | 0.112 | -0.360 | **0.019** | -0.297 | 0.056 | -0.143 | 0.366 |
| ACC_sup_R | -0.133 | 0.400 | -0.116 | 0.466 | -0.246 | 0.116 | -0.184 | 0.245 | -0.100 | 0.530 | -0.176 | 0.264 |

#####

## Table 3(2). Spearman correlation analysis of ipsilesional thalamic subfield covariance with N_ACC

| Targets | CeM | | VM | | VLp | | Pc | | Pt | |
| --- | --- | --- | --- | --- | --- | --- | --- | --- | --- | --- |
|  | r | p | r | p | r | p | r | p | r | p |
| IFGorb_R | 0.036 | 0.821 | 0.156 | 0.323 | 0.169 | 0.284 | -0.002 | 0.990 | 0.070 | 0.661 |
| OFCant_R | 0.126 | 0.425 | 0.358 | **0.020** | 0.161 | 0.308 | 0.079 | 0.619 | 0.160 | 0.311 |
| OFCpost_L | -0.021 | 0.894 | 0.203 | 0.198 | 0.031 | 0.845 | -0.202 | 0.199 | 0.003 | 0.987 |
| OFCpost_R | -0.005 | 0.974 | 0.144 | 0.363 | 0.145 | 0.360 | -0.059 | 0.712 | 0.020 | 0.900 |
| MCC_R | -0.248 | 0.114 | -0.119 | 0.453 | -0.181 | 0.252 | -0.351 | **0.023** | -0.429 | **0.005** |
| CAL_L | -0.041 | 0.794 | 0.002 | 0.988 | 0.016 | 0.921 | -0.032 | 0.842 | -0.232 | 0.140 |
| CAL_R | -0.161 | 0.309 | -0.125 | 0.430 | -0.031 | 0.847 | -0.067 | 0.672 | -0.307 | **0.048** |
| CUN_L | -0.072 | 0.652 | 0.109 | 0.492 | 0.038 | 0.809 | 0.043 | 0.786 | -0.168 | 0.289 |
| CUN_R | -0.113 | 0.475 | 0.144 | 0.363 | 0.042 | 0.793 | -0.017 | 0.916 | -0.153 | 0.335 |
| LING_R | -0.154 | 0.331 | -0.031 | 0.848 | -0.108 | 0.495 | -0.188 | 0.234 | -0.194 | 0.217 |
| SOG_L | -0.121 | 0.447 | 0.043 | 0.787 | -0.026 | 0.873 | -0.052 | 0.742 | -0.185 | 0.241 |
| SOG_R | -0.163 | 0.303 | -0.076 | 0.632 | -0.063 | 0.694 | -0.254 | 0.105 | -0.291 | 0.062 |
| PCL_R | -0.098 | 0.538 | 0.040 | 0.802 | 0.052 | 0.744 | -0.092 | 0.564 | -0.043 | 0.788 |
| CAU_R | -0.177 | 0.261 | -0.241 | 0.124 | -0.315 | **0.042** | -0.120 | 0.450 | -0.308 | **0.047** |
| PUT_R | -0.319 | **0.039** | -0.213 | 0.176 | -0.174 | 0.272 | -0.274 | 0.079 | -0.259 | 0.098 |
| PAL_R | -0.240 | 0.125 | -0.165 | 0.295 | -0.132 | 0.406 | -0.225 | 0.152 | -0.265 | 0.090 |
| ACC_sup_R | 0.062 | 0.697 | 0.079 | 0.617 | -0.007 | 0.966 | -0.137 | 0.387 | -0.149 | 0.346 |

##### Note: Black and bold represents P < 0.05, with underline represents P < 0.01.

## Table 4. Spearman correlation analysis of ipsilesional thalamic subfield covariance with N_RT

| Targets | MGN | | L_Sg | | VPL | | VLa | | VA | | VM | | PuL | | AV | |
| --- | --- | --- | --- | --- | --- | --- | --- | --- | --- | --- | --- | --- | --- | --- | --- | --- |
|  | r | p | r | p | r | p | r | p | r | p | r | p | r | p | r | p |
| IFGorb_R | 0.187 | 0.234 | 0.356 | **0.021** | 0.283 | 0.070 | 0.176 | 0.263 | 0.126 | 0.426 | 0.313 | **0.044** | 0.222 | 0.158 | 0.240 | 0.125 |
| OFCant_R | 0.109 | 0.489 | 0.237 | 0.131 | 0.045 | 0.776 | -0.016 | 0.919 | 0.061 | 0.703 | 0.036 | 0.820 | 0.319 | **0.040** | -0.029 | 0.856 |
| OFCpost_L | 0.030 | 0.851 | 0.236 | 0.133 | 0.069 | 0.663 | -0.097 | 0.540 | -0.074 | 0.642 | 0.117 | 0.458 | -0.069 | 0.663 | -0.087 | 0.584 |
| OFCpost_R | 0.178 | 0.259 | 0.351 | **0.023** | 0.205 | 0.192 | 0.094 | 0.554 | 0.108 | 0.497 | 0.276 | 0.077 | 0.102 | 0.518 | 0.210 | 0.181 |
| MCC_R | 0.239 | 0.127 | 0.293 | 0.060 | 0.105 | 0.507 | 0.113 | 0.474 | 0.179 | 0.255 | 0.157 | 0.321 | -0.083 | 0.599 | 0.266 | 0.089 |
| CAL_L | 0.063 | 0.690 | 0.121 | 0.443 | -0.062 | 0.695 | -0.014 | 0.929 | -0.131 | 0.407 | -0.108 | 0.496 | 0.019 | 0.905 | -0.045 | 0.778 |
| CAL_R | 0.113 | 0.477 | 0.116 | 0.463 | -0.012 | 0.940 | 0.120 | 0.447 | 0.047 | 0.768 | -0.027 | 0.867 | 0.032 | 0.843 | 0.125 | 0.428 |
| CUN_L | 0.048 | 0.761 | 0.202 | 0.199 | 0.080 | 0.613 | 0.008 | 0.963 | -0.035 | 0.824 | 0.012 | 0.938 | 0.259 | 0.097 | -0.129 | 0.413 |
| CUN_R | -0.046 | 0.774 | 0.214 | 0.174 | -0.020 | 0.898 | -0.087 | 0.581 | -0.147 | 0.353 | -0.015 | 0.927 | 0.170 | 0.280 | -0.174 | 0.269 |
| LING_R | 0.013 | 0.935 | 0.274 | 0.080 | -0.016 | 0.923 | -0.031 | 0.843 | 0.005 | 0.976 | 0.081 | 0.608 | 0.095 | 0.548 | 0.069 | 0.663 |
| SOG_L | 0.012 | 0.940 | 0.214 | 0.173 | -0.030 | 0.851 | -0.019 | 0.906 | 0.040 | 0.799 | 0.010 | 0.949 | 0.073 | 0.646 | -0.037 | 0.817 |
| SOG_R | 0.186 | 0.239 | 0.156 | 0.324 | -0.016 | 0.919 | 0.043 | 0.786 | 0.009 | 0.957 | -0.091 | 0.565 | 0.100 | 0.526 | -0.016 | 0.919 |
| PCL_R | 0.267 | 0.088 | 0.073 | 0.646 | 0.121 | 0.443 | -0.005 | 0.975 | 0.088 | 0.578 | 0.131 | 0.407 | 0.073 | 0.646 | 0.053 | 0.736 |
| CAU_R | 0.183 | 0.245 | 0.266 | 0.088 | 0.236 | 0.133 | 0.044 | 0.783 | 0.222 | 0.156 | 0.260 | 0.096 | 0.229 | 0.144 | 0.363 | **0.019** |
| PUT_R | 0.263 | 0.092 | 0.292 | 0.061 | 0.370 | **0.016** | 0.339 | **0.028** | 0.357 | **0.021** | 0.462 | **0.002** | 0.153 | 0.334 | 0.494 | **0.001** |
| PAL_R | 0.334 | **0.031** | 0.310 | **0.046** | 0.294 | 0.059 | 0.386 | **0.012** | 0.390 | **0.011** | 0.370 | **0.016** | 0.242 | 0.123 | 0.488 | **0.001** |
| ACC_sup_R | 0.043 | 0.786 | 0.114 | 0.470 | -0.037 | 0.816 | -0.030 | 0.852 | 0.003 | 0.984 | 0.091 | 0.566 | -0.089 | 0.574 | 0.193 | 0.221 |

##### Note: Black and bold represents P < 0.05, with underline represents P < 0.01.

## Table 5(1). Spearman correlation analysis of ipsilesional thalamic subfield covariance with S_ACC

| Targets | LGN | | MGN | | L_Sg | | VPL | | VLa | | PuA | | MDm | | Pf | | VAmc | | MDl | |
| --- | --- | --- | --- | --- | --- | --- | --- | --- | --- | --- | --- | --- | --- | --- | --- | --- | --- | --- | --- | --- |
|  | r | p | r | p | r | p | r | p | r | p | r | p | r | p | r | p | r | p | r | p |
| IFGorb_R | 0.051 | 0.750 | -0.342 | **0.027** | -0.150 | 0.345 | 0.120 | 0.450 | -0.137 | 0.388 | -0.287 | 0.066 | -0.164 | 0.298 | -0.019 | 0.906 | -0.143 | 0.365 | -0.254 | 0.105 |
| OFCant_R | 0.373 | **0.015** | -0.192 | 0.223 | -0.170 | 0.283 | 0.188 | 0.232 | 0.023 | 0.886 | -0.182 | 0.248 | 0.023 | 0.885 | 0.107 | 0.500 | 0.041 | 0.797 | -0.036 | 0.820 |
| OFCpost_L | 0.140 | 0.378 | -0.238 | 0.129 | -0.046 | 0.772 | 0.214 | 0.175 | 0.019 | 0.906 | -0.060 | 0.708 | 0.093 | 0.557 | -0.137 | 0.388 | 0.037 | 0.816 | 0.067 | 0.676 |
| OFCpost_R | 0.024 | 0.880 | -0.406 | **0.008** | -0.074 | 0.640 | 0.124 | 0.432 | -0.159 | 0.314 | -0.256 | 0.102 | -0.163 | 0.302 | 0.001 | 0.997 | -0.192 | 0.224 | -0.208 | 0.187 |
| MCC_R | 0.076 | 0.632 | -0.247 | 0.115 | -0.212 | 0.177 | -0.210 | 0.181 | -0.343 | **0.026** | -0.358 | **0.020** | -0.288 | 0.064 | -0.271 | 0.083 | -0.320 | **0.039** | -0.317 | **0.041** |
| CAL_L | 0.019 | 0.906 | -0.190 | 0.228 | -0.143 | 0.367 | 0.014 | 0.932 | -0.150 | 0.342 | -0.362 | **0.019** | -0.174 | 0.271 | -0.048 | 0.765 | -0.144 | 0.362 | -0.403 | **0.008** |
| CAL_R | -0.014 | 0.932 | -0.079 | 0.619 | -0.176 | 0.264 | -0.117 | 0.462 | -0.227 | 0.148 | -0.392 | **0.010** | -0.256 | 0.102 | -0.115 | 0.470 | -0.139 | 0.381 | -0.414 | **0.007** |
| CUN_L | 0.025 | 0.877 | -0.198 | 0.209 | -0.097 | 0.541 | -0.046 | 0.773 | -0.191 | 0.226 | -0.408 | **0.007** | -0.175 | 0.267 | -0.045 | 0.779 | -0.171 | 0.279 | -0.352 | **0.022** |
| CUN_R | 0.171 | 0.280 | 0.018 | 0.908 | -0.147 | 0.352 | 0.088 | 0.581 | -0.129 | 0.417 | -0.240 | 0.126 | -0.191 | 0.225 | 0.026 | 0.872 | -0.154 | 0.330 | -0.271 | 0.083 |
| LING_R | 0.065 | 0.683 | -0.111 | 0.486 | -0.068 | 0.667 | -0.013 | 0.938 | -0.255 | 0.104 | -0.266 | 0.088 | -0.272 | 0.082 | -0.063 | 0.691 | -0.280 | 0.073 | -0.323 | **0.037** |
| SOG_L | 0.182 | 0.249 | -0.148 | 0.350 | -0.128 | 0.418 | -0.055 | 0.729 | -0.080 | 0.615 | -0.127 | 0.423 | 0.065 | 0.682 | 0.042 | 0.791 | -0.010 | 0.951 | -0.032 | 0.840 |
| SOG_R | 0.204 | 0.196 | -0.058 | 0.716 | -0.373 | **0.015** | -0.136 | 0.389 | -0.111 | 0.483 | -0.138 | 0.384 | -0.113 | 0.478 | -0.128 | 0.419 | -0.142 | 0.372 | -0.173 | 0.273 |
| PCL_R | 0.431 | **0.004** | 0.097 | 0.541 | -0.183 | 0.246 | 0.221 | 0.159 | 0.232 | 0.139 | 0.069 | 0.663 | 0.140 | 0.378 | 0.086 | 0.587 | 0.163 | 0.304 | 0.142 | 0.370 |
| CAU_R | -0.257 | 0.101 | -0.159 | 0.315 | -0.054 | 0.735 | -0.316 | **0.041** | -0.384 | **0.012** | -0.227 | 0.149 | -0.278 | 0.075 | -0.313 | **0.044** | -0.395 | **0.010** | -0.278 | 0.075 |
| PUT_R | -0.097 | 0.543 | -0.130 | 0.412 | -0.252 | 0.108 | -0.114 | 0.473 | -0.260 | 0.096 | -0.116 | 0.466 | -0.355 | **0.021** | -0.201 | 0.202 | -0.357 | **0.020** | -0.302 | 0.052 |
| PAL_R | 0.059 | 0.711 | -0.217 | 0.168 | -0.341 | 0.027 | -0.045 | 0.777 | -0.246 | 0.117 | -0.195 | 0.215 | -0.312 | **0.044** | -0.186 | 0.239 | -0.290 | 0.063 | -0.310 | **0.046** |
| ACC_sup_R | 0.042 | 0.794 | -0.129 | 0.414 | -0.076 | 0.632 | -0.089 | 0.577 | -0.216 | 0.170 | -0.294 | 0.058 | -0.266 | 0.088 | -0.304 | 0.051 | -0.170 | 0.283 | -0.334 | **0.031** |

##### Note: Black and bold represents P < 0.05, with underline represents P < 0.01.

## Table 5(2). Spearman correlation analysis of ipsilesional thalamic subfield covariance with S_ACC

| Targets | VA | | MV | | CeM | | VM | | CL | | VLp | | Pc | | Pt | | AV | |
| --- | --- | --- | --- | --- | --- | --- | --- | --- | --- | --- | --- | --- | --- | --- | --- | --- | --- | --- |
|  | r | p | r | p | r | p | r | p | r | p | r | p | r | p | r | p | r | p |
| IFGorb_R | -0.248 | 0.113 | -0.054 | 0.735 | -0.091 | 0.567 | 0.148 | 0.351 | -0.035 | 0.827 | 0.061 | 0.700 | -0.145 | 0.359 | -0.026 | 0.870 | -0.120 | 0.449 |
| OFCant_R | 0.015 | 0.924 | -0.126 | 0.426 | 0.011 | 0.945 | 0.193 | 0.220 | 0.013 | 0.934 | 0.136 | 0.390 | 0.016 | 0.921 | 0.221 | 0.159 | 0.023 | 0.887 |
| OFCpost_L | -0.011 | 0.946 | -0.038 | 0.810 | 0.067 | 0.674 | 0.244 | 0.120 | 0.077 | 0.630 | 0.104 | 0.514 | 0.014 | 0.932 | 0.195 | 0.216 | 0.135 | 0.395 |
| OFCpost_R | -0.291 | 0.062 | -0.017 | 0.913 | -0.122 | 0.441 | 0.124 | 0.435 | 0.070 | 0.658 | 0.032 | 0.839 | -0.109 | 0.491 | 0.038 | 0.812 | -0.090 | 0.572 |
| MCC_R | -0.439 | **0.004** | -0.193 | 0.221 | -0.259 | 0.097 | -0.215 | 0.171 | -0.161 | 0.309 | -0.208 | 0.187 | -0.260 | 0.097 | -0.153 | 0.332 | -0.328 | **0.034** |
| CAL_L | -0.111 | 0.486 | -0.255 | 0.103 | -0.122 | 0.441 | 0.000 | 1.000 | -0.078 | 0.623 | 0.030 | 0.852 | -0.006 | 0.972 | -0.052 | 0.745 | -0.049 | 0.760 |
| CAL_R | -0.221 | 0.161 | -0.136 | 0.392 | -0.136 | 0.392 | -0.184 | 0.245 | -0.059 | 0.710 | -0.121 | 0.445 | -0.009 | 0.956 | -0.092 | 0.563 | -0.209 | 0.184 |
| CUN_L | -0.118 | 0.459 | -0.338 | **0.029** | -0.230 | 0.143 | -0.005 | 0.976 | -0.113 | 0.475 | -0.098 | 0.536 | 0.018 | 0.912 | -0.065 | 0.682 | -0.132 | 0.407 |
| CUN_R | -0.126 | 0.428 | -0.112 | 0.479 | -0.060 | 0.705 | 0.027 | 0.867 | 0.137 | 0.386 | -0.049 | 0.757 | 0.088 | 0.580 | 0.092 | 0.562 | -0.098 | 0.538 |
| LING_R | -0.265 | 0.089 | -0.142 | 0.370 | -0.178 | 0.260 | -0.066 | 0.677 | 0.090 | 0.571 | -0.127 | 0.422 | -0.089 | 0.573 | 0.003 | 0.984 | -0.035 | 0.826 |
| SOG_L | -0.034 | 0.830 | -0.216 | 0.169 | -0.100 | 0.530 | -0.150 | 0.344 | -0.013 | 0.938 | -0.080 | 0.613 | 0.116 | 0.464 | 0.093 | 0.559 | -0.069 | 0.664 |
| SOG_R | -0.136 | 0.390 | -0.202 | 0.199 | -0.135 | 0.393 | -0.182 | 0.250 | 0.116 | 0.466 | -0.024 | 0.883 | -0.095 | 0.550 | -0.131 | 0.409 | 0.020 | 0.899 |
| PCL_R | 0.114 | 0.474 | 0.111 | 0.485 | 0.126 | 0.426 | 0.180 | 0.255 | 0.062 | 0.696 | 0.281 | 0.071 | 0.045 | 0.779 | 0.214 | 0.174 | 0.185 | 0.241 |
| CAU_R | -0.390 | **0.011** | -0.332 | **0.032** | -0.357 | **0.020** | -0.310 | **0.045** | -0.320 | **0.039** | -0.364 | **0.018** | -0.261 | 0.095 | -0.347 | **0.025** | -0.302 | 0.052 |
| PUT_R | -0.393 | **0.010** | -0.028 | 0.861 | -0.224 | 0.153 | -0.162 | 0.306 | -0.070 | 0.658 | -0.142 | 0.371 | -0.354 | **0.021** | -0.255 | 0.103 | -0.268 | 0.087 |
| PAL_R | -0.309 | **0.047** | -0.050 | 0.754 | -0.209 | 0.183 | -0.125 | 0.430 | 0.010 | 0.950 | -0.085 | 0.593 | -0.247 | 0.115 | -0.172 | 0.277 | -0.205 | 0.193 |
| ACC_sup_R | -0.332 | **0.032** | 0.049 | 0.758 | -0.016 | 0.921 | -0.038 | 0.811 | -0.050 | 0.752 | -0.123 | 0.440 | -0.196 | 0.213 | 0.061 | 0.701 | -0.118 | 0.455 |

##### Note: Black and bold represents P < 0.05, with underline represents P < 0.01.

## Table 6. Spearman correlation analysis of ipsilesional thalamic subfield covariance with S_RT

| Targets | MGN | | L_Sg | | VPL | | MDl | | VM | | AV | | LP | | LD | |
| --- | --- | --- | --- | --- | --- | --- | --- | --- | --- | --- | --- | --- | --- | --- | --- | --- |
|  | r | p | r | p | r | p | r | p | r | p | r | p | r | p | r | p |
| IFGorb_R | 0.257 | 0.101 | 0.476 | **0.002** | 0.369 | **0.017** | 0.016 | 0.920 | 0.389 | **0.011** | 0.168 | 0.288 | 0.147 | 0.351 | 0.123 | 0.435 |
| OFCant_R | 0.282 | 0.071 | 0.365 | **0.018** | 0.169 | 0.283 | -0.026 | 0.869 | 0.183 | 0.245 | 0.075 | 0.638 | -0.006 | 0.969 | 0.026 | 0.869 |
| OFCpost_L | 0.003 | 0.986 | 0.276 | 0.077 | 0.083 | 0.602 | -0.064 | 0.687 | 0.132 | 0.405 | -0.114 | 0.471 | -0.029 | 0.857 | -0.121 | 0.444 |
| OFCpost_R | 0.163 | 0.300 | 0.402 | **0.009** | 0.269 | 0.085 | 0.028 | 0.859 | 0.310 | **0.047** | 0.134 | 0.398 | 0.168 | 0.288 | 0.137 | 0.384 |
| MCC_R | 0.169 | 0.282 | 0.297 | 0.056 | -0.069 | 0.663 | -0.130 | 0.412 | -0.003 | 0.985 | 0.006 | 0.968 | -0.014 | 0.932 | -0.181 | 0.251 |
| CAL_L | 0.118 | 0.455 | 0.244 | 0.120 | -0.042 | 0.790 | -0.222 | 0.157 | -0.141 | 0.372 | -0.158 | 0.317 | -0.144 | 0.362 | -0.151 | 0.338 |
| CAL_R | 0.134 | 0.395 | 0.239 | 0.127 | 0.034 | 0.832 | -0.049 | 0.759 | -0.030 | 0.848 | 0.021 | 0.893 | 0.077 | 0.628 | -0.046 | 0.772 |
| CUN_L | 0.083 | 0.602 | 0.227 | 0.148 | 0.087 | 0.584 | -0.040 | 0.803 | 0.007 | 0.965 | -0.132 | 0.405 | -0.040 | 0.800 | -0.136 | 0.390 |
| CUN_R | 0.009 | 0.958 | 0.281 | 0.072 | 0.003 | 0.984 | -0.297 | 0.056 | -0.027 | 0.868 | -0.212 | 0.177 | -0.169 | 0.285 | -0.263 | 0.093 |
| LING_R | 0.024 | 0.878 | 0.325 | **0.037** | -0.089 | 0.573 | -0.130 | 0.409 | -0.006 | 0.970 | 0.002 | 0.992 | -0.049 | 0.757 | -0.128 | 0.418 |
| SOG_L | -0.062 | 0.694 | 0.214 | 0.172 | -0.127 | 0.421 | -0.044 | 0.780 | -0.034 | 0.832 | -0.075 | 0.636 | -0.024 | 0.882 | -0.132 | 0.402 |
| SOG_R | 0.122 | 0.439 | 0.210 | 0.181 | -0.132 | 0.405 | -0.335 | **0.031** | -0.186 | 0.237 | -0.128 | 0.417 | -0.319 | **0.040** | -0.350 | **0.024** |
| PCL_R | 0.338 | 0.029 | 0.138 | 0.382 | 0.102 | 0.519 | -0.173 | 0.272 | 0.172 | 0.276 | 0.056 | 0.726 | -0.089 | 0.573 | 0.024 | 0.879 |
| CAU_R | 0.232 | 0.139 | 0.291 | 0.062 | 0.124 | 0.431 | 0.005 | 0.973 | 0.197 | 0.210 | 0.319 | **0.040** | 0.065 | 0.682 | 0.039 | 0.808 |
| PUT_R | 0.257 | 0.101 | 0.388 | **0.012** | 0.233 | 0.138 | -0.025 | 0.873 | 0.339 | **0.028** | 0.275 | 0.079 | 0.101 | 0.523 | 0.007 | 0.965 |
| PAL_R | 0.327 | **0.035** | 0.426 | **0.005** | 0.210 | 0.181 | 0.046 | 0.771 | 0.305 | 0.050 | 0.300 | 0.054 | 0.122 | 0.442 | 0.002 | 0.989 |
| ACC_sup_R | 0.111 | 0.482 | 0.277 | 0.076 | -0.135 | 0.394 | -0.074 | 0.643 | -0.022 | 0.893 | -0.012 | 0.938 | 0.014 | 0.931 | -0.027 | 0.868 |

##### Note: Black and bold represents P < 0.05, with underline represents P < 0.01.

## Table 7. Spearman correlation analysis of ipsilesional thalamic subfield covariance with FMA

| Targets | MGN | | L_Sg | | PuL | | CL | | VLp | | AV | | LP | | LD | |
| --- | --- | --- | --- | --- | --- | --- | --- | --- | --- | --- | --- | --- | --- | --- | --- | --- |
|  | r | p | r | p | r | p | r | p | r | p | r | p | r | p | r | p |
| IFGorb_R | -0.126 | 0.428 | -0.260 | 0.096 | -0.139 | 0.381 | 0.413 | **0.007** | 0.241 | 0.124 | 0.415 | **0.006** | 0.182 | 0.249 | 0.347 | **0.025** |
| OFCant_R | -0.098 | 0.537 | -0.209 | 0.184 | -0.101 | 0.525 | 0.305 | 0.050 | 0.077 | 0.630 | 0.176 | 0.266 | 0.165 | 0.298 | 0.325 | **0.036** |
| OFCpost_L | -0.307 | **0.048** | -0.215 | 0.172 | -0.143 | 0.366 | 0.173 | 0.274 | -0.170 | 0.283 | 0.181 | 0.252 | 0.141 | 0.373 | 0.218 | 0.166 |
| OFCpost_R | -0.163 | 0.304 | -0.333 | **0.031** | -0.085 | 0.594 | 0.328 | **0.034** | 0.188 | 0.232 | 0.386 | **0.012** | 0.205 | 0.192 | 0.305 | **0.049** |
| MCC_R | -0.247 | 0.115 | -0.028 | 0.861 | -0.344 | **0.026** | 0.098 | 0.537 | -0.150 | 0.343 | -0.018 | 0.910 | 0.087 | 0.584 | 0.083 | 0.602 |
| CAL_L | -0.075 | 0.639 | 0.354 | **0.022** | -0.339 | **0.028** | 0.044 | 0.780 | -0.158 | 0.316 | -0.026 | 0.871 | -0.114 | 0.471 | 0.021 | 0.897 |
| CAL_R | 0.030 | 0.852 | 0.359 | **0.019** | -0.324 | **0.037** | -0.032 | 0.840 | -0.093 | 0.558 | -0.015 | 0.927 | -0.103 | 0.518 | 0.056 | 0.726 |
| CUN_L | 0.186 | 0.238 | 0.490 | **0.001** | -0.241 | 0.125 | 0.081 | 0.610 | -0.315 | **0.042** | -0.085 | 0.595 | -0.126 | 0.427 | 0.139 | 0.382 |
| CUN_R | 0.023 | 0.886 | 0.342 | **0.027** | -0.283 | 0.069 | 0.064 | 0.687 | -0.295 | 0.058 | 0.034 | 0.832 | -0.040 | 0.804 | 0.203 | 0.198 |
| LING_R | -0.153 | 0.334 | 0.205 | 0.193 | -0.108 | 0.497 | -0.058 | 0.715 | -0.065 | 0.683 | -0.030 | 0.849 | 0.019 | 0.908 | -0.070 | 0.658 |
| SOG_L | 0.194 | 0.218 | 0.351 | **0.023** | -0.283 | 0.069 | -0.053 | 0.738 | -0.272 | 0.082 | -0.169 | 0.285 | -0.317 | **0.041** | -0.019 | 0.904 |
| SOG_R | 0.075 | 0.638 | 0.296 | 0.057 | -0.198 | 0.208 | -0.038 | 0.810 | -0.062 | 0.698 | -0.006 | 0.972 | -0.053 | 0.737 | 0.078 | 0.622 |
| PCL_R | -0.018 | 0.909 | 0.002 | 0.990 | -0.318 | **0.040** | 0.135 | 0.393 | 0.036 | 0.822 | 0.077 | 0.629 | 0.028 | 0.860 | 0.227 | 0.148 |
| CAU_R | -0.095 | 0.550 | 0.248 | 0.113 | 0.171 | 0.278 | 0.114 | 0.472 | -0.225 | 0.151 | -0.048 | 0.765 | 0.153 | 0.333 | 0.066 | 0.679 |
| PUT_R | -0.069 | 0.663 | -0.066 | 0.679 | 0.042 | 0.794 | 0.165 | 0.298 | -0.014 | 0.929 | 0.172 | 0.277 | 0.172 | 0.275 | 0.111 | 0.483 |
| PAL_R | -0.089 | 0.573 | -0.092 | 0.563 | 0.003 | 0.988 | 0.206 | 0.190 | -0.007 | 0.968 | 0.235 | 0.135 | 0.246 | 0.116 | 0.133 | 0.402 |
| ACC_sup_R | -0.225 | 0.153 | -0.004 | 0.980 | -0.178 | 0.260 | 0.195 | 0.215 | 0.047 | 0.767 | -0.034 | 0.832 | 0.207 | 0.189 | -0.013 | 0.937 |

##### Note: Black and bold represents P < 0.05, with underline represents P < 0.01.

## Table 8(1). Spearman correlation analysis of contralesional thalamic subfield covariance with RAVLT_SR

| Targets | MGN | | PuI | | PuM | | VPL | | CM | | VLa | | PuA | | Pf | |
| --- | --- | --- | --- | --- | --- | --- | --- | --- | --- | --- | --- | --- | --- | --- | --- | --- |
|  | r | p | r | p | r | p | r | p | r | p | r | p | r | p | r | p |
| IFGorb_R | -0.012 | 0.940 | 0.405 | **0.008** | 0.305 | **0.049** | 0.349 | **0.024** | 0.270 | 0.084 | 0.227 | 0.149 | 0.119 | 0.453 | 0.212 | 0.178 |
| OFCant_R | -0.159 | 0.314 | 0.456 | **0.002** | 0.483 | **0.001** | 0.359 | **0.020** | 0.431 | **0.004** | 0.309 | **0.046** | 0.347 | **0.024** | 0.431 | **0.004** |
| OFCpost_R | 0.008 | 0.959 | 0.408 | **0.007** | 0.295 | 0.058 | 0.404 | **0.008** | 0.289 | 0.064 | 0.264 | 0.092 | 0.177 | 0.263 | 0.186 | 0.240 |
| CAU_R | -0.043 | 0.785 | 0.187 | 0.235 | 0.100 | 0.528 | -0.013 | 0.937 | 0.095 | 0.552 | 0.092 | 0.561 | -0.167 | 0.292 | 0.211 | 0.180 |
| PUT_R | -0.249 | 0.112 | 0.052 | 0.746 | -0.133 | 0.400 | -0.114 | 0.471 | -0.129 | 0.414 | -0.109 | 0.492 | -0.352 | **0.022** | -0.023 | 0.887 |
| PAL_R | -0.329 | **0.033** | 0.050 | 0.752 | -0.108 | 0.495 | -0.083 | 0.600 | -0.137 | 0.388 | -0.094 | 0.556 | -0.292 | 0.061 | -0.013 | 0.935 |

##### Note: Black and bold represents P < 0.05, with underline represents P < 0.01.

## Table 8(2). Spearman correlation analysis of contralesional thalamic subfield covariance with RAVLT_SR

| Targets | VAmc | | CeM | | MV(Re) | | VM | | Pt | | Pc | | LP | | LD | |
| --- | --- | --- | --- | --- | --- | --- | --- | --- | --- | --- | --- | --- | --- | --- | --- | --- |
|  | r | p | r | p | r | p | r | p | r | p | r | p | r | p | r | p |
| IFGorb_R | 0.129 | 0.417 | 0.183 | 0.246 | 0.143 | 0.367 | 0.408 | **0.007** | 0.371 | **0.016** | 0.268 | 0.087 | 0.188 | 0.234 | 0.271 | 0.083 |
| OFCant_R | 0.349 | **0.023** | 0.364 | **0.018** | 0.306 | **0.048** | 0.517 | **0.001** | 0.355 | **0.021** | 0.404 | **0.008** | 0.311 | **0.045** | 0.452 | **0.003** |
| OFCpost_R | 0.132 | 0.407 | 0.183 | 0.247 | 0.151 | 0.338 | 0.405 | **0.008** | 0.397 | **0.009** | 0.242 | 0.123 | 0.170 | 0.282 | 0.247 | 0.115 |
| CAU_R | 0.086 | 0.589 | 0.092 | 0.564 | 0.095 | 0.551 | 0.078 | 0.625 | 0.013 | 0.937 | 0.109 | 0.493 | 0.163 | 0.303 | 0.036 | 0.820 |
| PUT_R | -0.214 | 0.174 | -0.119 | 0.451 | -0.166 | 0.293 | -0.095 | 0.552 | -0.002 | 0.992 | -0.153 | 0.334 | 0.143 | 0.368 | 0.033 | 0.837 |
| PAL_R | -0.206 | 0.192 | -0.095 | 0.548 | -0.121 | 0.446 | -0.047 | 0.768 | 0.002 | 0.989 | -0.138 | 0.382 | 0.189 | 0.230 | 0.074 | 0.644 |

##### Note: Black and bold represents P < 0.05, with underline represents P < 0.01.

## Table 9. Spearman correlation analysis of contralesional thalamic subfield covariance with RAVLT_LR

| Targets | MGN | | PuI | | Pt | |
| --- | --- | --- | --- | --- | --- | --- |
|  | r | p | r | p | r | p |
| IFGorb_R | -0.138 | 0.383 | 0.502 | **0.001** | 0.320 | **0.039** |
| OFCant_R | -0.229 | 0.145 | 0.409 | **0.007** | 0.190 | 0.229 |
| OFCpost_R | -0.101 | 0.525 | 0.465 | **0.002** | 0.292 | 0.061 |
| CAU_R | -0.233 | 0.137 | 0.188 | 0.232 | -0.032 | 0.839 |
| PUT_R | -0.288 | 0.065 | 0.238 | 0.129 | -0.025 | 0.877 |
| PAL_R | -0.360 | **0.019** | 0.231 | 0.142 | -0.043 | 0.787 |

##### Note: Black and bold represents P < 0.05, with underline represents P < 0.01.

## Table 10. Spearman correlation analysis of contralesional thalamic subfield covariance with N_ACC

| Targets | MGN | | PuA | | CL | | Pt | | LD | |
| --- | --- | --- | --- | --- | --- | --- | --- | --- | --- | --- |
|  | r | p | r | p | r | p | r | p | r | p |
| IFGorb_R | -0.247 | 0.115 | 0.156 | 0.325 | 0.182 | 0.249 | 0.039 | 0.807 | 0.105 | 0.507 |
| OFCant_R | -0.341 | **0.027** | 0.049 | 0.759 | 0.398 | **0.009** | 0.103 | 0.519 | 0.324 | **0.037** |
| OFCpost_R | -0.277 | 0.076 | 0.160 | 0.312 | 0.192 | 0.223 | 0.052 | 0.742 | 0.132 | 0.405 |
| CAU_R | -0.145 | 0.361 | 0.115 | 0.468 | 0.089 | 0.574 | -0.325 | **0.036** | -0.075 | 0.638 |
| PUT_R | -0.160 | 0.311 | 0.280 | 0.072 | -0.247 | 0.115 | -0.134 | 0.397 | -0.358 | **0.020** |
| PAL_R | -0.183 | 0.247 | 0.334 | **0.031** | -0.223 | 0.157 | -0.118 | 0.456 | -0.305 | **0.049** |

##### Note: Black and bold represents P < 0.05, with underline represents P < 0.01.

##### Table 11. Spearman correlation analysis of contralesional thalamic subfield covariance with N_RT

| Targets | MDm | | CeM | | VA | | LP | | LD | |
| --- | --- | --- | --- | --- | --- | --- | --- | --- | --- | --- |
|  | r | p | r | p | r | p | r | p | r | p |
| IFGorb_R | -0.210 | 0.181 | -0.086 | 0.587 | 0.114 | 0.472 | -0.114 | 0.471 | -0.106 | 0.504 |
| OFCant_R | -0.363 | **0.019** | -0.310 | **0.046** | 0.060 | 0.706 | -0.325 | **0.036** | -0.439 | **0.004** |
| OFCpost_R | -0.184 | 0.243 | -0.051 | 0.749 | 0.164 | 0.297 | -0.055 | 0.731 | -0.039 | 0.805 |
| CAU_R | -0.070 | 0.657 | -0.060 | 0.705 | 0.114 | 0.470 | -0.254 | 0.105 | -0.236 | 0.133 |
| PUT_R | 0.145 | 0.360 | 0.163 | 0.300 | 0.290 | 0.063 | -0.052 | 0.742 | 0.021 | 0.897 |
| PAL_R | 0.131 | 0.408 | 0.192 | 0.222 | 0.318 | **0.041** | -0.067 | 0.672 | 0.028 | 0.862 |

##### Note: Black and bold represents P < 0.05, with underline represents P < 0.01.

## Table 12(1). Spearman correlation analysis of contralesional thalamic subfield covariance with S_ACC

| Targets | MGN | | VPL | | CM | | VLa | | MDm | | VAmc | | CeM | | VA | |
| --- | --- | --- | --- | --- | --- | --- | --- | --- | --- | --- | --- | --- | --- | --- | --- | --- |
|  | r | p | r | p | r | p | r | p | r | p | r | p | r | p | r | p |
| IFGorb_R | -0.385 | **0.012** | -0.215 | 0.172 | -0.071 | 0.654 | -0.160 | 0.313 | 0.218 | 0.166 | -0.012 | 0.941 | -0.002 | 0.989 | -0.193 | 0.221 |
| OFCant_R | -0.250 | 0.111 | 0.142 | 0.369 | 0.225 | 0.151 | 0.073 | 0.647 | 0.112 | 0.480 | 0.143 | 0.366 | 0.021 | 0.896 | -0.084 | 0.597 |
| OFCpost_R | -0.401 | **0.009** | -0.159 | 0.314 | 0.005 | 0.977 | -0.096 | 0.544 | 0.214 | 0.174 | 0.034 | 0.832 | 0.033 | 0.836 | -0.126 | 0.425 |
| CAU_R | -0.096 | 0.546 | -0.261 | 0.095 | -0.237 | 0.130 | -0.460 | **0.002** | -0.419 | **0.006** | -0.395 | **0.010** | -0.329 | **0.034** | -0.486 | **0.001** |
| PUT_R | -0.220 | 0.162 | -0.388 | **0.011** | -0.308 | **0.047** | -0.414 | **0.006** | -0.024 | 0.882 | -0.314 | **0.043** | -0.166 | 0.293 | -0.330 | **0.033** |
| PAL_R | -0.282 | 0.071 | -0.332 | **0.032** | -0.292 | 0.061 | -0.361 | **0.019** | 0.027 | 0.866 | -0.257 | 0.101 | -0.125 | 0.431 | -0.263 | 0.093 |

##### Note: Black and bold represents P < 0.05, with underline represents P < 0.01.

## Table 12(2). Spearman correlation analysis of contralesional thalamic subfield covariance with S_ACC

| Targets | MV | | CL | | PuL | | Pt | | AV | | Pc | | VLp | | LD | |
| --- | --- | --- | --- | --- | --- | --- | --- | --- | --- | --- | --- | --- | --- | --- | --- | --- |
|  | r | p | r | p | r | p | r | p | r | p | r | p | r | p | r | p |
| IFGorb_R | -0.001 | 0.995 | -0.068 | 0.669 | -0.374 | **0.015** | -0.124 | 0.436 | -0.133 | 0.400 | -0.045 | 0.777 | -0.192 | 0.224 | -0.083 | 0.602 |
| OFCant_R | -0.107 | 0.502 | 0.071 | 0.653 | -0.259 | 0.098 | 0.140 | 0.376 | -0.103 | 0.516 | 0.122 | 0.442 | 0.097 | 0.542 | 0.046 | 0.771 |
| OFCpost_R | 0.073 | 0.648 | -0.094 | 0.552 | -0.359 | **0.020** | -0.077 | 0.630 | -0.074 | 0.640 | 0.024 | 0.879 | -0.154 | 0.332 | -0.008 | 0.959 |
| CAU_R | -0.332 | **0.032** | -0.314 | **0.043** | 0.103 | 0.515 | -0.333 | **0.031** | -0.385 | **0.012** | -0.235 | 0.134 | -0.408 | **0.007** | -0.420 | **0.006** |
| PUT_R | -0.011 | 0.947 | -0.112 | 0.479 | 0.007 | 0.963 | -0.392 | **0.010** | -0.152 | 0.336 | -0.306 | **0.049** | -0.400 | **0.009** | -0.205 | 0.192 |
| PAL_R | 0.020 | 0.901 | -0.061 | 0.700 | -0.064 | 0.689 | -0.343 | **0.026** | -0.142 | 0.372 | -0.279 | 0.074 | -0.324 | **0.036** | -0.149 | 0.347 |

##### Note: Black and bold represents P < 0.05, with underline represents P < 0.01.

## Table 13. Spearman correlation analysis of contralesional thalamic subfield covariance with S_RT

| Targets | MV | |
| --- | --- | --- |
|  | r | p |
| IFGorb_R | 0.121 | 0.445 |
| OFCant_R | -0.200 | 0.204 |
| OFCpost_R | 0.117 | 0.461 |
| CAU_R | -0.099 | 0.530 |
| PUT_R | 0.317 | **0.041** |
| PAL_R | 0.337 | **0.030** |

##### Note: Black and bold represents P < 0.05


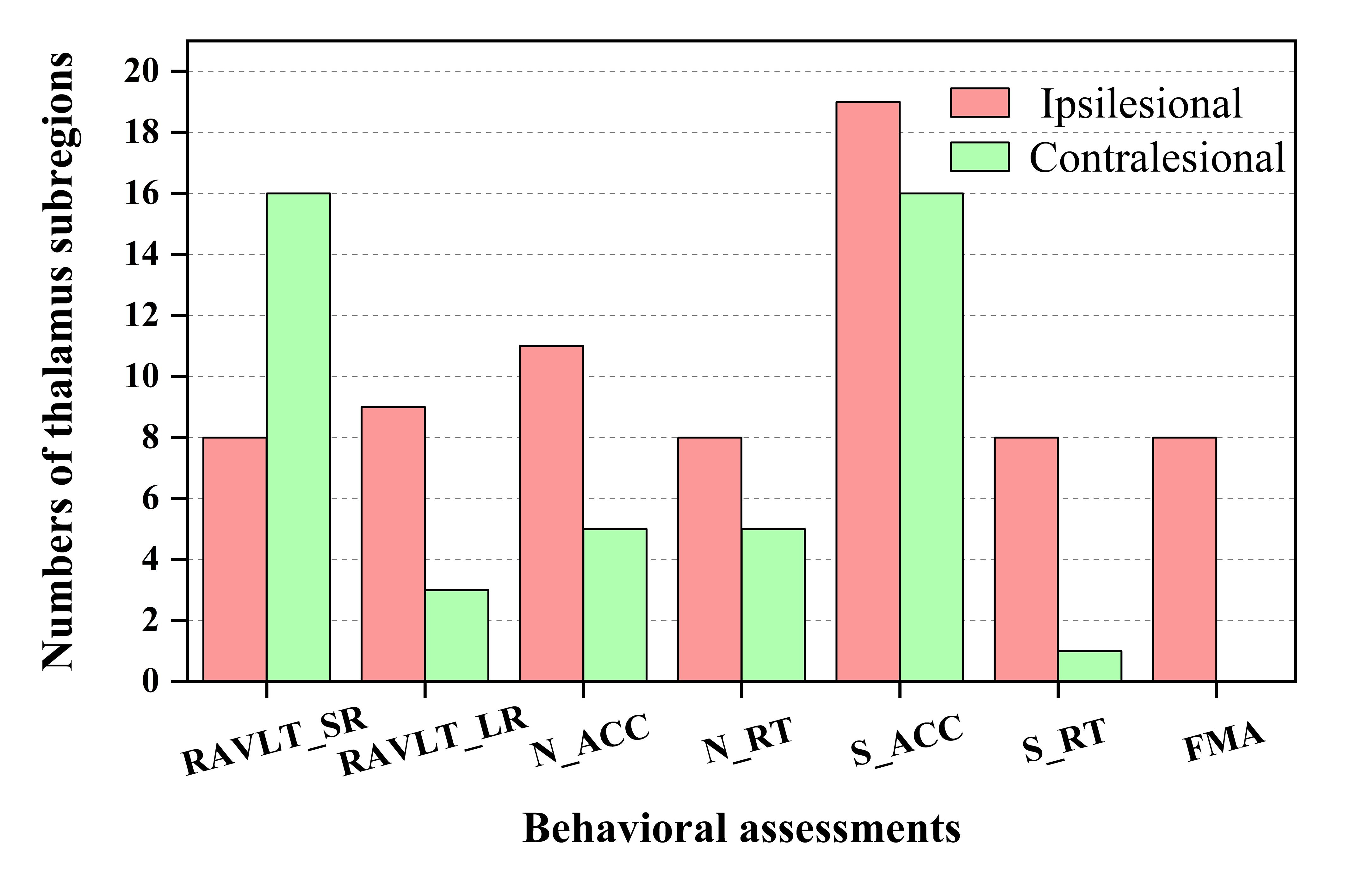


**Figure 4.** The total number of thalamic subfields that are associated with different behavioral assessments. Significance is based on uncorrected P < 0.05.


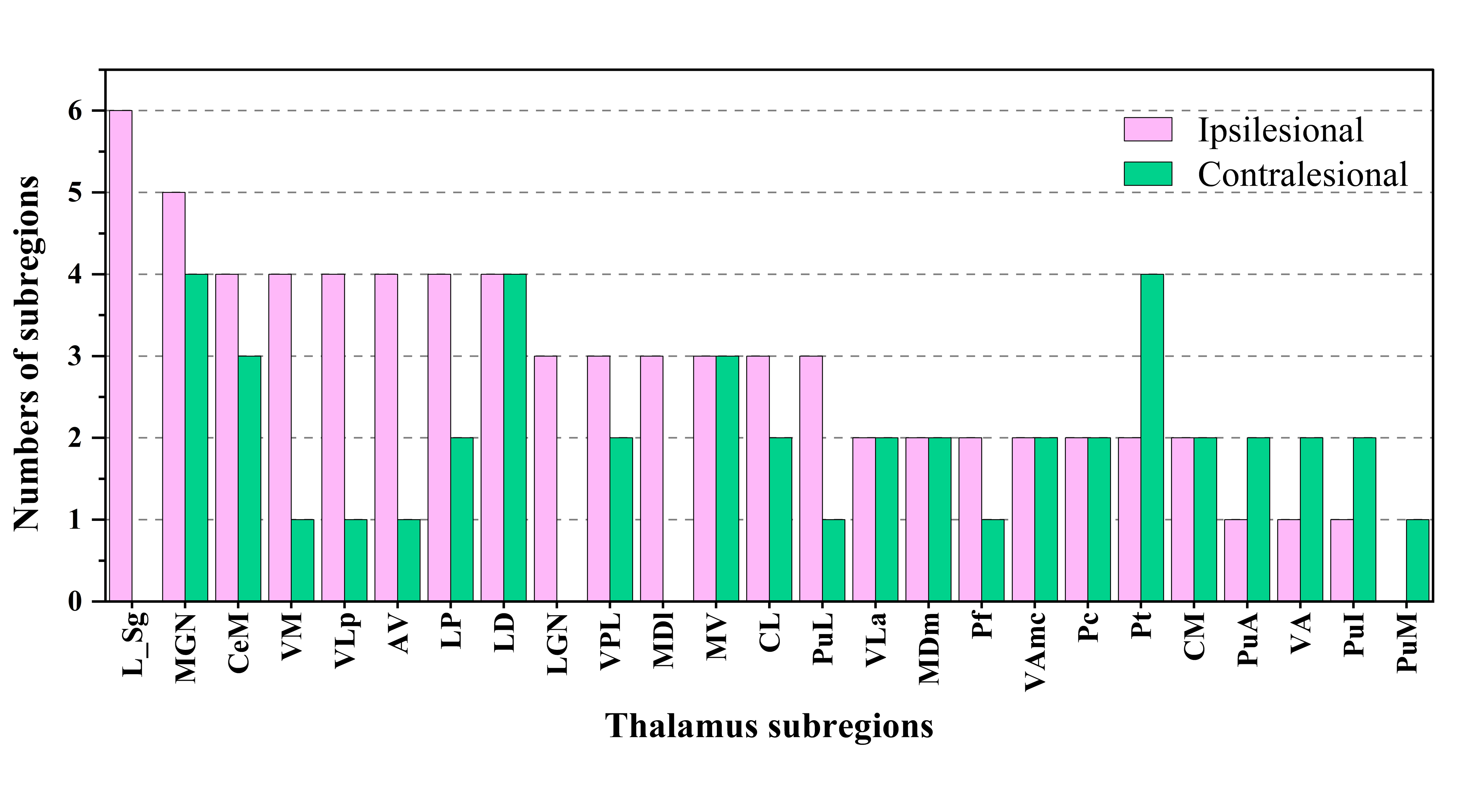


**Figure 5.** The numbers of each thalamic subfield associated with behavioral assessments. Significance is based on uncorrected P < 0.05.


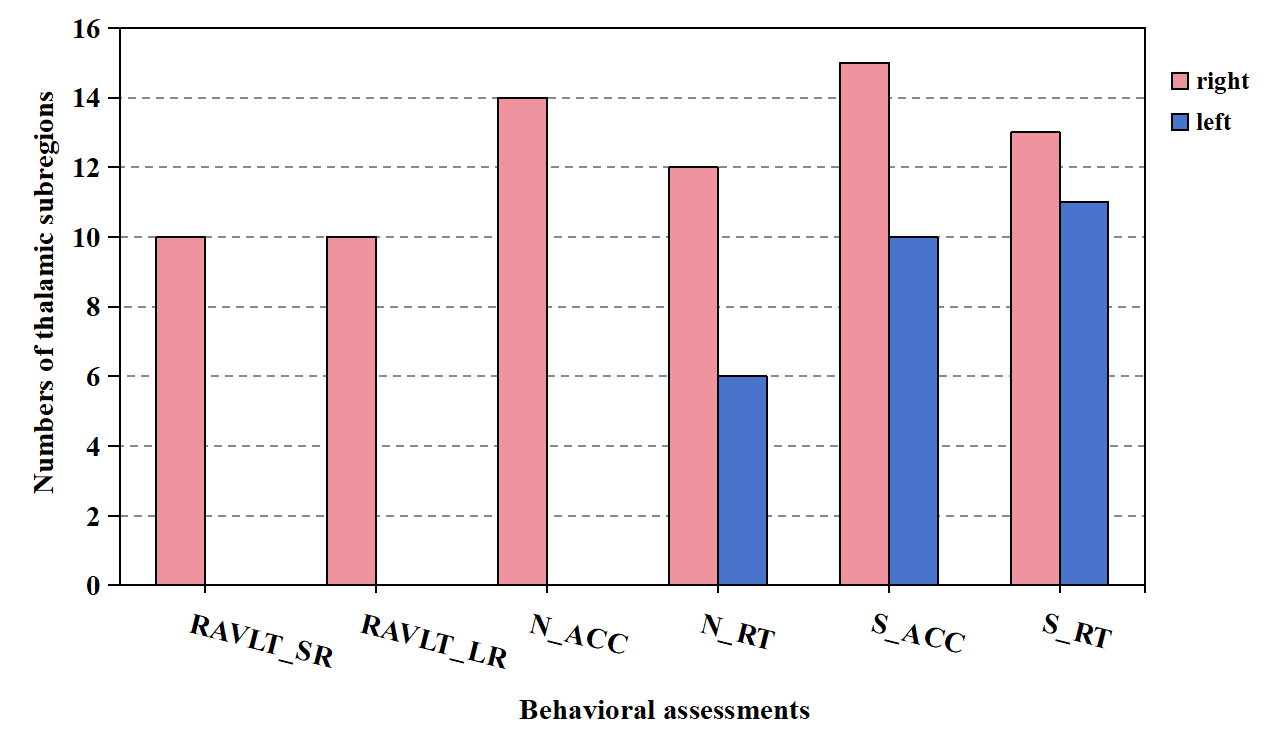


**Figure 6.** The total number of thalamic subregions (NC group) that are associated with different behavioral assessments. Significance is based on uncorrected P < 0.05.


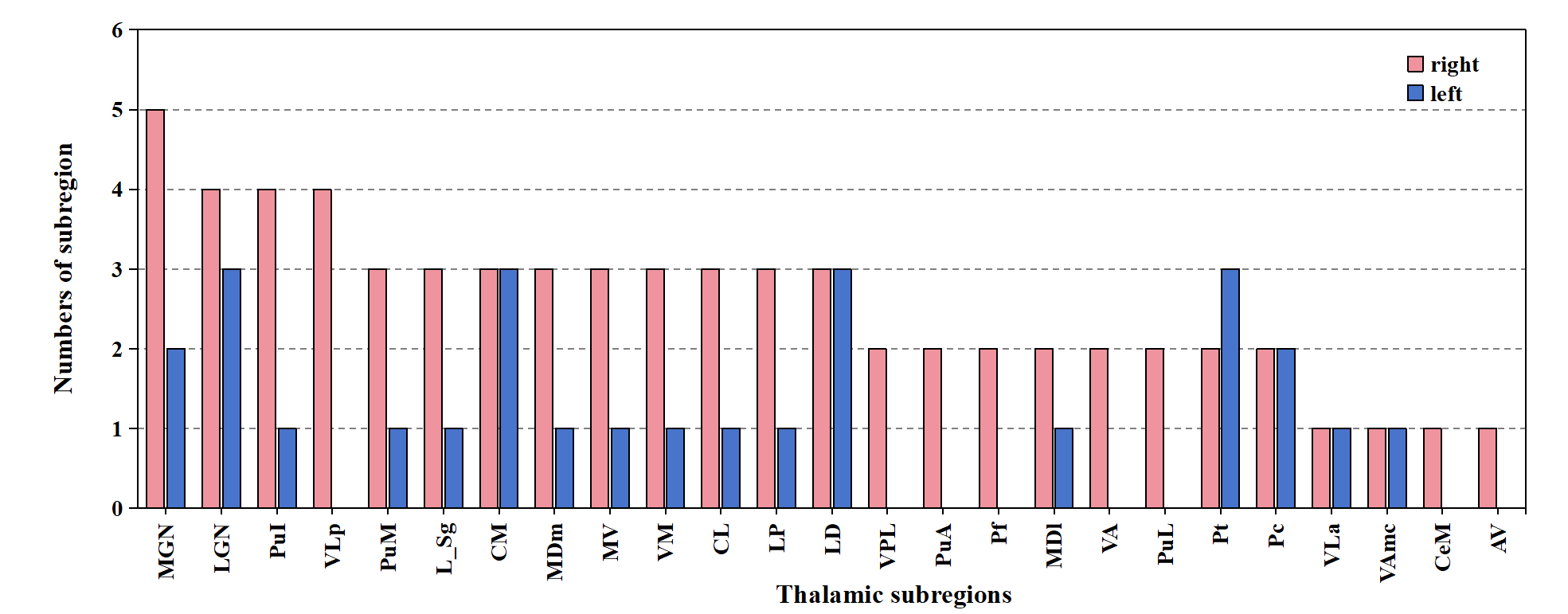


**Figure 7.** The numbers of each thalamic subregions (NC group) associated with behavioral assessments. Significance is based on uncorrected P < 0.05.


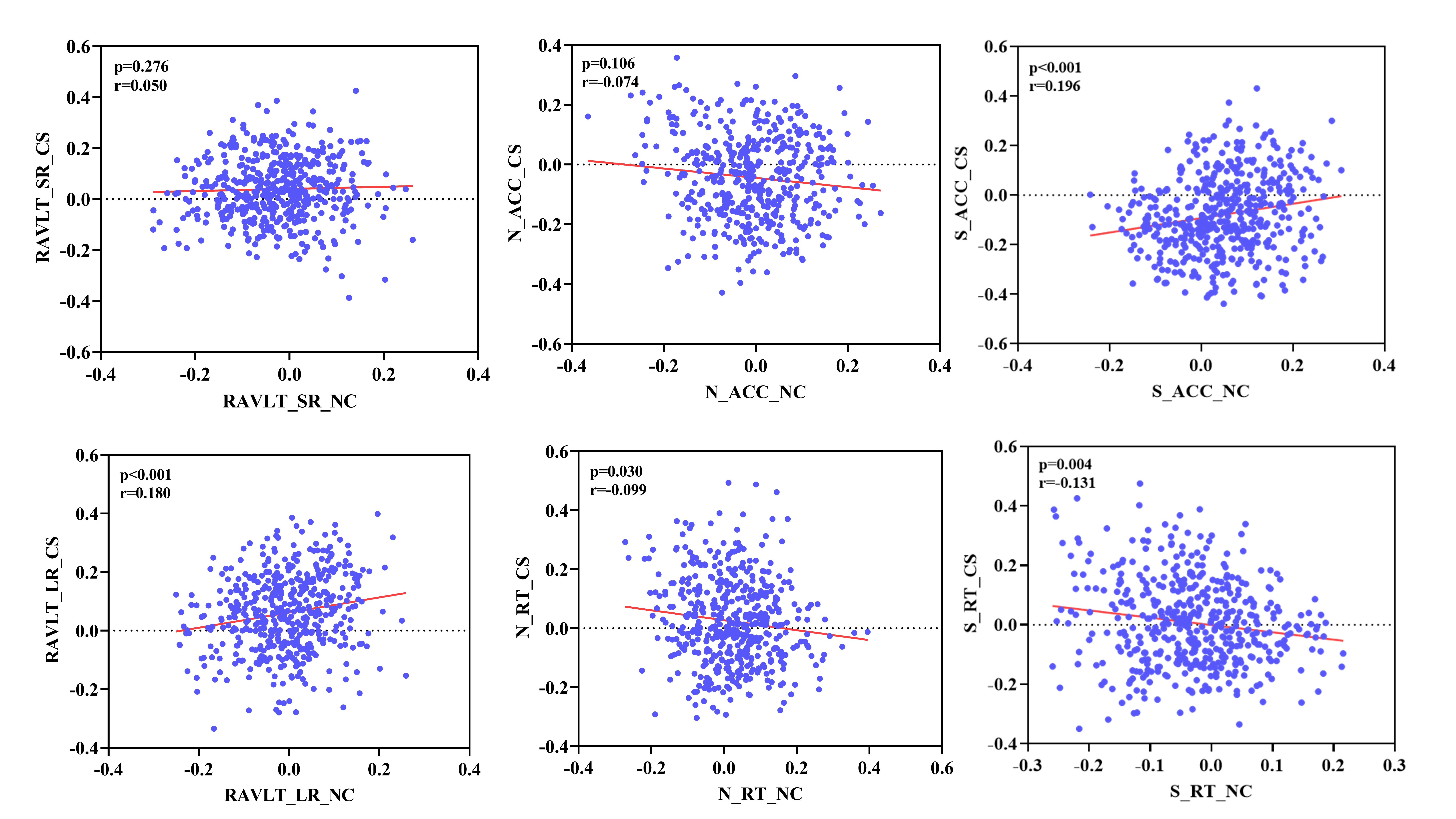


**Figure 8.** The association of the correlation coefficient between the right thalamic SCN and clinical metrics was compared across the CS and NC groups. Significance is based on P < 0.05.


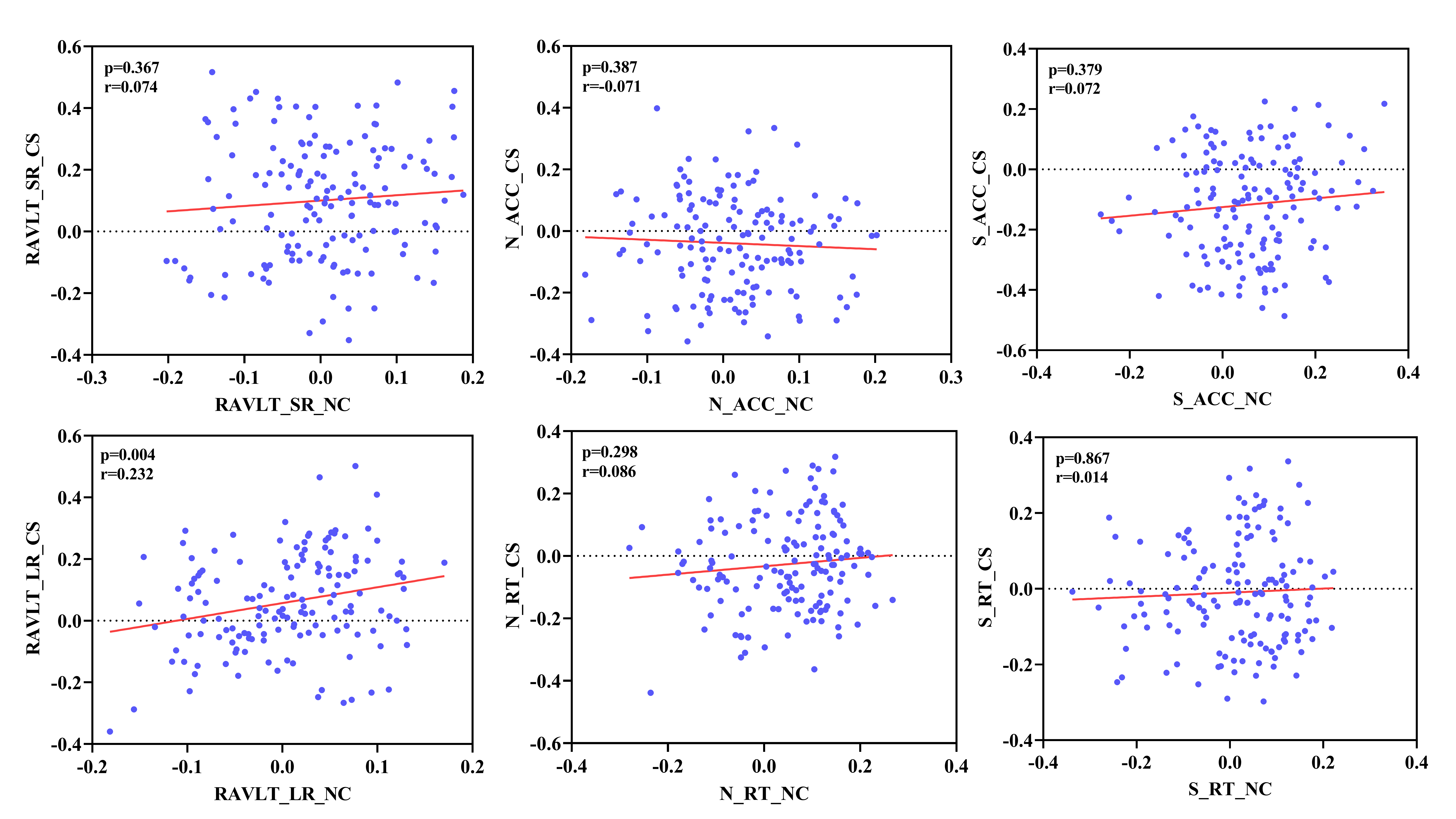


**Figure 9.** The association of the correlation coefficient between the left thalamic SCN and clinical metrics was compared across the CS and NC groups. Significance is based on P < 0.05.
